# Transcriptomic elucidation of Dahuang-Huanglian in promoting white adipose browning in high-fat diet-induced obese rats

**DOI:** 10.3389/fendo.2025.1652703

**Published:** 2025-10-08

**Authors:** Ruiyao Zhang, Yu Zhang, Xi Xi, Pengcheng Du, Chao Guo, Yanying Zhang, Bing Song, Xiaoyan Xu, Zhitao Ni, Yongfeng Wang, Min Bai

**Affiliations:** ^1^ Gansu University of Chinese Medicine, Lanzhou, China; ^2^ Gansu Provincial Technical Center for Laboratory Animals, Lanzhou, China; ^3^ Affiliated Hospital of Gansu University of Chinese Medicine, Lanzhou, China; ^4^ Gansu Medical College, Pingliang, China; ^5^ Ningxia Medical University, Yinchuan, China

**Keywords:** transcriptomics, Dahuang-Huanglian, AMPK, white adipose tissue browning, obesity

## Abstract

**Objective:**

Dahuang (*Rhei Radix et Rhizoma*)-Huanglian (*Coptidis Rhizoma*) (DHHL), has been shown to effectively treat obesity caused by dietary irregularities. Nevertheless, the fundamental process driving this phenomenon has yet to be elucidated.

**Methods:**

The chemical constituents of DHHL were analyzed using UPLC-MS/MS. An obesity model was established in rats by high-fat diet (HFD) induction and verified accordingly. Obese rats were administered various doses of DHHL. Detect and record the metabolic indicators of rats in each group. Transcriptomic analysis was used to evaluate the influence of DHHL on gene expression in obese rats. H&E staining and transmission electron microscopy (TEM) was used to observe the morphology of adipocytes. Immunohistochemistry (IHC), fluorescent immunohistochemistry (FIHC), and Western blotting (WB) were performed to detect protein expression levels.

**Results:**

The chemical constituents of DHHL medicinal materials were identified and analyzed using UPLC-MS/MS. Total ion chromatograms (TIC) were acquired in both positive and negative ion modes. Pie charts were generated to illustrate the abundance distribution and quantitative proportion of different components. HFD feeding induced significant increases in body weight and FBG in rats, elevated serum triglycerides (TG) and free fatty acids (FFA) levels, and promoted hypertrophy and hyperplasia of adipose tissue, while also disrupting glucose metabolism. DHHL treatment significantly improved body weight, FBG, glucose uptake capacity, and insulin sensitivity in obese rats. It also reduced blood lipid levels and lipid accumulation in a dose-dependent manner. Transcriptomic sequencing revealed that the anti-obesity effects of DHHL were closely associated with the upregulation of thermogenesis-related gene expression. KEGG pathway enrichment analysis indicated that DHHL may exert regulatory effects through pathways such as AMPK, PPAR, and PI3K. TEM observations demonstrated that DHHL increased mitochondrial numbers within adipocytes of obese rats. Molecular analyses further showed that DHHL upregulated the expression of thermogenesis-associated proteins—including PPARγ, PRDM16, and UCP1—thereby promoting the browning of white adipose tissue (WAT). Moreover, DHHL enhanced the expression levels of AMPK, SIRT1, and PGC-1α.

**Conclusions:**

DHHL effectively ameliorates HFD-induced obesity in rats, and its therapeutic mechanism is closely associated with the activation of the AMPK/SIRT1/PGC-1α signaling pathway, which promotes the browning of WAT.

## 1 Introduction

A chronic metabolic condition with multiple contributing factors, obesity develops when prolonged discrepancies exist between caloric consumption and energy utilization, leading to surplus energy storage within bodily tissues (1). With the continuous improvement of economic conditions and living standards, the global prevalence of obesity has shown an upward trend year by year (2). The pathogenesis of obesity is complex and influenced by multiple factors, and current intervention strategies have various limitations. Therefore, there is an urgent need to explore more rational and feasible approaches for its prevention and treatment. Prolonged energy surplus leads to excessive synthesis of triglycerides, which accumulate in adipose tissues and are histologically characterized by hypertrophy and hyperplasia of adipocytes (3). In obesity pathogenesis, the gradual expansion of adipose depots represents a fundamental pathophysiological mechanism. Thus, strategies aimed at reducing lipid accumulation and promoting the decomposition and utilization of triglycerides are considered effective means of combating obesity.

Traditional Chinese Medicine (TCM) has demonstrated significant efficacy in the treatment of chronic metabolic diseases such as obesity. Dahuang(*Rhei Radix et Rhizoma*)-Huanglian(*Coptidis Rhizoma*) (DHHL) are the principal herbal components of the classical TCM formula Da Huang Huang Lian Xie Xin Tang (Rhubarb and Coptis Heart-Draining Decoction), and both herbs have been shown to exert anti-obesity effects. *Rhei Radix et Rhizoma* is a medicinal herb with a long history of traditional use and demonstrates a variety of potential therapeutic benefits, including anti-hyperlipidemic effects among its broad spectrum of pharmacological activities (4). Literature reviews have indicated that various species of *Rhei Radix et Rhizoma* demonstrate remarkable improvements in metabolic disorders, particularly in the treatment of obesity, through mechanisms such as inhibiting key enzymes involved in lipogenesis, regulating glucose homeostasis, and enhancing energy metabolism (5). Following its treatment, significant amelioration of metabolic parameters was observed in diet-induced obese rats, including reduced serum levels of TG and TC, as well as improved liver function (6). Studies have also shown that dietary supplementation with *Rhei Radix et Rhizoma* may help prevent metabolic diseases such as obesity and type 2 diabetes mellitus (T2DM) induced by a diet rich in lipids and carbohydrates, potentially through mechanisms involving modulation of the gut microbiota (7). *Rhei Radix et Rhizoma* is rich in anthraquinone compounds. Experimental studies have revealed that these anthraquinones exhibit significant regulatory effects on lipid metabolism. Beyond modulating genes associated with lipid metabolism, their hypolipidemic actions are closely linked to energy metabolism pathways such as AMPK and PPAR signaling (8). Research has directly demonstrated that *Rhei Radix et Rhizoma* extract exerts its anti-obesity and metabolic-improving effects by targeting the SIRT6 and AMPK pathways in adipose tissue, thereby promoting energy expenditure (9). Similarly, *Coptidis Rhizoma* is another traditional medicinal herb with broad biological benefits, showing remarkable therapeutic potential against metabolic disorders such as obesity and T2DM. Obesity is characterized by a chronic low-grade inflammatory state. Studies have indicated that intervention with *Coptidis Rhizoma* not only reduces fat accumulation and blood glucose levels in high-fat diet-fed mice but also significantly ameliorates adipose tissue inflammation (10).A meta-analysis focusing on adjunctive therapy with Coptis preparations for T2DM has indicated that its application plays an important role in improving glycemic control in diabetic patients (11). Another randomized controlled clinical study demonstrated that berberine, the primary active constituent of Coptidis Rhizoma, exerts favorable effects on body composition, blood pressure, and the expression of adipokine genes associated with metabolic risk in obese patients (12). Fundamental research has further confirmed that the therapeutic benefits of Coptidis Rhizoma in alleviating obesity and metabolic disorders are closely associated with its ability to enhance lipolysis and promote energy metabolism. In summary, the combination of DHHL promising therapeutic potential for the treatment of obesity and related metabolic disorders (13). Moreover, the db/db mouse model, when treated with DHHL, exhibited noteworthy enhancements in weight management and glucose-lipid metabolic indices, as documented in our earlier research (14). However, whether DHHL can ameliorate obesity caused solely by excessive energy intake, as well as the underlying therapeutic mechanisms, remains unclear.

Transcriptomics is a pivotal discipline that investigates gene expression at the RNA molecular level within tissues or cells. Its core objective lies in the systematic analysis of the entire set of RNA molecules transcribed under specific spatial and temporal conditions in biological samples, followed by their integrative or categorical analysis. This approach holds substantial value for exploring gene expression levels and functions (15). Transcriptomic techniques not only elucidate the dynamic changes in gene expression during the onset and progression of diseases but also facilitate the identification of therapeutic targets and regulatory pathways.

In the human body, adipose tissue can be classified into brown adipose tissue (BAT) and white adipose tissue (WAT). WAT primarily functions in energy storage, thermal insulation, and mechanical cushioning, whereas BAT dissipates energy by increasing thermogenesis. A distinguishing structural feature of BAT is its high mitochondrial density and abundant expression of uncoupling protein 1 (UCP1), which is critical for thermogenic activity (16). Recent studies have revealed that WAT, particularly subcutaneous white adipose tissue, contains scattered clusters of cells that can be induced to form beige/brown-like adipocytes, whose structural and functional properties closely resemble those of BAT (17). When subjected to particular environmental cues (e.g., reduced temperature) or pharmacological agents (e.g., small-molecule compounds), these plastic cells can exhibit phenotypic conversion toward a brown adipocyte-like state—a physiological adaptation designated as white adipose tissue browning. This transformation is considered a key mechanism to increase energy expenditure and reduce fat accumulation (18). In research on WAT browning, inguinal white adipose tissue (iWAT) is frequently employed as a representative depot. Upon browning, iWAT exhibits a marked upregulation of the thermogenic protein UCP1 and an increase in mitochondrial content (19), ultimately enhancing thermogenesis and promoting lipid catabolism. Investigating strategies to promote WAT browning may therefore offer novel approaches to counter the obesity epidemic.

AMP-activated protein kinase (AMPK) serves as a central metabolic sensor in cells. Structurally, it comprises three subunits: the α subunit functions as the catalytic core, while the β and γ subunits serve as regulatory components (20). Once activated, AMPK can enhance energy expenditure by promoting thermogenesis, thereby ameliorating lipid accumulation. Numerous studies have shown that AMPK activation alleviates WAT accumulation and induces its browning (21, 22). Moreover, the browning effects of cold exposure and β3-adrenergic receptor stimulation are also partially mediated by the activation of the AMPK pathway (23, 24). Silent information regulator 1 (SIRT1), a deacetylase, also functions as a cellular energy sensor and plays a key role in improving metabolic function (25). SIRT1 has been closely associated with white adipose tissue browning; upon activation, it promotes browning by upregulating the expression of genes related to this process (26). Peroxisome proliferator-activated receptor gamma coactivator 1-alpha (PGC-1α) is crucial in maintaining mitochondrial homeostasis and regulating energy metabolism (27). Studies have shown that AMPK can promote the expression and activation of thermogenic proteins such as UCP1 by stimulating the SIRT1/PGC-1α pathway, thereby enhancing the browning of WAT (28, 29).

In this study, a high-fat diet (HFD)-induced rat model of obesity was established. After treatment with DHHL, body weight and glucose and lipid metabolic indices were assessed to evaluate DHHL’s anti-obesity effects. Subsequently, transcriptomic sequencing of iWAT was performed, and potential therapeutic targets and pathways were further validated. These findings aim to provide experimental evidence supporting the use of DHHL in the treatment of obesity.

## 2 Materials and methods

### 2.1 Materials

High-fat diet (D12451, comprising 45% fat, 35% carbohydrates, and 20% protein) purchased from Shenyang Maohua Biotechnology Co., Ltd. The authenticated crude drugs of Rhei Radix et Rhizoma (202210653–3) and Coptidis Rhizoma (231002) were procured from the Affiliated Hospital of Gansu University of Chinese Medicine. Metformin hydrochloride tablets (ABZ4625) was obtained from Sino-American Shanghai Squibb Pharmaceuticals Ltd.

Rat INS ELISA Kit (YJ985241) Rat FFA ELISA KIT (YJ524122) were purchased from Shanghai Yuanju Biotechnology Center. p-AMPKα (14099) antibody SIRT1 (27523) antibody PGC-1α (27583) antibody were purchased from Signalwayantibody Co., Ltd. PPARγ (YT3836) antibody was obtained from ImmunoWay Biotechnology Company. PRDM16 (DF13303) antibody was purchased from affinity biosciences. UCP1 (GB112174) antibody was obtained from Wuhan Servicebio Technology Co., Ltd. β-tubulin (380628) Goat-anti-Mouse IgG-HRP (511103) were purchased from Chengdu Zen-Bioscience Co., Ltd.

### 2.2 Drugs and reagents

The Dahuang-Huanglian herbal preparation was processed according to Chinese Pharmacopoeia specifications and prepared by the Pharmaceutical Preparation Laboratory of Gansu University of Chinese Medicine Affiliated Hospital. Based on our research group’s previous studies, a dosage of DHHL was set at 10 g for each component, in a 1:1 ratio (14). Using a multifunctional extraction tank, the first extraction involved soaking crude drugs in 10-fold volume of distilled water for 30 minutes, followed by conventional decoction twice (40 minutes per cycle). The filtered solutions were combined and concentrated to achieve 1g crude drug per mL. After high-temperature sterilization and cooling, the extract was stored at 4°C. For experimental use, the stock solution was diluted proportionally prior to administration.

### 2.3 Animals and treatments

Eighty SPF-grade male SD rats, aged 7 weeks old, were obtained by the Gansu University of Chinese Medicine (Laboratory animal production license No. SYXK(Gan)2020-0001, Ethical Review Approval No. SY2024-232). The rats were fed with standard rodent food and water and under stable light-dark (12h-12h) cycles and constant room temperature. The Animal Welfare Law of China is strictly observed during animal experiments, and the supervision and inspection of the Laboratory Animal Ethics Committee is accepted at all times.

After one week of acclimatization feeding, all rats were randomly divided into two groups using a random number table method: the normal diet (ND) group (*n* = 16) fed standard chow and the high-fat diet (HFD) group (*n* = 64) fed fat-enriched chow. Obesity modeling was assessed following 8 weeks of HFD feeding, with rats exceeding 20% of the ND group’s average body weight defined as obese model animals (30), while non-qualified rats were excluded. To validate successful modeling, 6 rats each from ND and HFD groups were randomly selected for additional evaluation. Obese rats confirmed as successful models continued HFD feeding and were randomly allocated into five groups (*n* = 8 each): Model group, metformin (Met) group, low-dose Dahuang-Huanglian (LDHHL) group, medium-dose Dahuang-Huanglian (MDHHL) group, and high-dose Dahuang-Huanglian (HDHHL) group, while ND rats served as the Control group (*n* = 8). The HDHHL, MDHHL, and LDHHL groups received daily gavage of 3.6 g/kg, 1.8 g/kg, and 0.9 g/kg DHHL extract respectively. The Met group was administered 0.18 g/kg/day metformin hydrochloride suspension according to pharmaceutical guidelines. Both Control and Model groups received equivalent volumes of saline daily. All treatments were administered once daily for 4 consecutive weeks.

### 2.4 Examination of DHHL extract by UPLC-MS/MS

100 μL liquid sample was added to a 1.5 mL centrifuge tube with 400 μL solution (acetonitrile: methanol = 1:1(v:v)) containing four internal standards (0.02 mg/mL L-2-chlorophenylalanine, etc.) to extract metabolites. The samples were mixed by vortex for 30 s and low-temperature sonicated for 30 min (5°C, 40 KHz). The samples were placed at -20°C for 30 min to precipitate the proteins. Then the samples were centrifuged for 15 min (4°C, 13000 g). The supernatant was removed and blown dry under nitrogen. The sample was then re-solubilized with 100 μL solution (acetonitrile: water = 1:1) and extracted by low-temperature ultrasonication for 5 min (5°C, 40 KHz), followed by centrifugation at 13000 g and 4°C for 10 min. The supernatant was transferred to sample vials for LC-MS/MS analysis.

The LC-MS/MS analysis of sample was conducted on a UHPLC-Orbitrap Exploris 240 system equipped with an ACQUITY HSS T3 column (100 mm × 2.1 mm i.d., 1.8 μm; Waters, USA) at Majorbio Bio-Pharm Technology Co. Ltd. (Shanghai, China). The mobile phases consisted of 0.1% formic acid in water:acetonitrile (2:98, v/v) (solvent A) and 0.1% formic acid in acetonitrile (solvent B). The flow rate was 0.40 mL/min and the column temperature was 40°C. The injection volume was 5 μL (31).

MS conditions: The UPLC system was coupled to a UHPLC-Orbitrap Exploris 240 system Mass Spectrometer equipped with an electrospray ionization (ESI) source operating in positive mode and negative mode. The optimal conditions were set as followed: source temperature at 400°C; sheath gas flow rate at 40 arb; Aux gas flow rate at 10 arb; ion-spray voltage floating (ISVF) at -2800V in negative mode and 3500V in positive mode, respectively; Normalized collision energy, 20-40-60V rolling for MS/MS. Data acquisition was performed with the Data Dependent Acquisition (DDA) mode. The detection was carried out over a mass range of 70–1050 m/z.

Progenesis QI (Waters Corporation, Milford, USA) software was used for peak extraction, retention time correction and peak alignment of raw data, and metabolites were identified by matching HMDB, Metlin and self-built database; After screening valid variables by 80% rule, fill missing values with minimum value, normalize sum and eliminate variables with RSD>30% of QC samples. PCA/OPLS-DA model analysis (7 cross validation) was performed by using R language ropls package after log10 transformation to screen differential metabolites with VIP>1 and p<0.05. Thanks for the pathway annotation based on KEGG database and Fisher exact test enrichment analysis via Python’s scipy.stats package, all performed on the Majorbio.com platform.

### 2.5 Body weight and Lee’s index

Body weight measurements were determined at weekly intervals across experimental cohorts. Prior to tissue collection under anesthesia, final body weight and nose-to-anus length were quantified for each animal. Lee’s adiposity index was calculated using the formula: [cube root of body weight (g) × 1000]/body length (cm).

### 2.6 Fasting blood glucose

Fasting blood glucose (FBG) monitoring was performed weekly across experimental groups. Following 12-hour fasting with free water access, blood samples were obtained via caudal vein puncture using aseptic techniques. Glucose quantification was conducted immediately with Roche Accu-Chek monitoring systems and manufacturer-specified test strips. Post-sampling wound care involved prompt antisepsis using povidone-iodine solution, followed by hemostatic compression. All procedures maintained circadian consistency, with measurements conducted between 8:00-10:00 AM to minimize diurnal variation interference.

### 2.7 Free fatty acid, fasting serum insulin and HOMA-IR

Serum free fatty acids (FFA) and fasting serum insulin (FINS) levels were measured using ELISA kits. The homeostasis model assessment of insulin resistance (HOMA-IR) was calculated as follows: HOMA-IR = [FINS (μU/mL) × FBG (mmol/L)]/22.5.

### 2.8 Intraperitoneal glucose tolerance test and insulin tolerance test

Rats in each group were fasted for 12 h prior to the intraperitoneal glucose tolerance test (IPGTT). Following body weight measurement, 50% glucose solution was intraperitoneally administered at a dose of 2 g/kg. Blood samples were obtained from the tail vein at 0, 15, 30, 60, and 120 min post-injection for glucose determination using a glucometer. IPGTT curves were plotted, and the area under the curve (AUC) was calculated through trapezoidal integration.

For the insulin tolerance test (ITT), animals were fasted for 6 h before receiving intraperitoneal insulin injection (0.75 U/kg) following body weight measurement. Blood glucose levels were assessed at identical time intervals as IPGTT to construct ITT curves and compute AUC values.

### 2.9 Serum biochemical tests

Serum biochemical parameters including triglycerides (TG), total cholesterol (TC), low-density lipoprotein cholesterol (LDL-c), and high-density lipoprotein cholesterol (HDL-c) were quantified using a fully automatic biochemical analyzer (Roche cobas c 311).

### 2.10 H&E staining

Adipose tissue specimens were immersion-fixed in 4% paraformaldehyde, subsequently processed through dehydration and paraffin embedding, and sectioned at 5 μm thickness. Tissue sections were subjected to hematoxylin and eosin (H&E) staining for comprehensive histological evaluation.

### 2.11 Transcriptomic sequencing

Total RNA was extracted from rat adipose tissue using TRIzol reagent (Invitrogen), with optimized tissue mass (20–50 mg) adjusted for low RNA abundance. Following homogenization (60 Hz, 60 s) and phase separation with chloroform, RNA was precipitated with isopropanol and washed with 75% ethanol. RNA integrity was confirmed by Agilent 2100 Bioanalyzer (RNA 6000 Nano kit) and concentration quantified via NanoDrop 2000. Libraries were constructed from ≥1 μg total RNA using the NEBNext Ultra II Directional RNA Library Prep Kit for Illumina, involving poly(A) mRNA enrichment, fragmentation, cDNA synthesis, end repair/adapter ligation, and size selection (400–500 bp). Library quality was assessed by Agilent 2100 High Sensitivity DNA Kit and quantified by QPCR (StepOnePlus). Multiplexed libraries were sequenced on the Illumina platform (PE150). Raw data were filtered by removing adapter sequences and low-quality reads (Q20 cutoff). Clean reads were aligned to the rat reference genome (rn6) using HISAT2 v2.0.5, followed by gene expression quantification (HTSeq v0.9.1, FPKM normalization) and differential expression analysis (DESeq v1.20.0; |log_2_FC| >1, p < 0.05). Functional enrichment of differentially expressed genes was analyzed via GO (topGO) and KEGG (clusterProfiler v3.4.4).

### 2.12 Fluorescent immunohistochemistry

Paraffin-embedded tissue sections underwent deparaffinization with xylene and graded ethanol series. Antigen retrieval was performed using EDTA-based antigen retrieval buffer followed by blocking with bovine serum albumin. Sequential incubations with primary and secondary antibodies were conducted, accompanied by nuclear counterstaining with DAPI. Autofluorescence was eliminated using commercial quenching reagent before fluorescence-preserving mounting. Specimens were examined under fluorescence microscopy, and fluorescence intensity was quantified through ImageJ analysis software.

### 2.13 Western blotting

The thoroughly homogenized tissues were lysed in RIPA lysis buffer supplemented with protease and phosphatase inhibitors on ice for 30 min. After centrifugation at 12000 × g for 15 min, the supernatant was collected for protein quantification using BCA Protein Assay Kit. Equal amounts of protein samples mixed with loading buffer were denatured by boiling at 100°C for 10 min. Proteins were separated through SDS-PAGE and electrophoretically transferred to nitrocellulose membranes. Membranes were blocked with 5% skim milk for 1 h, followed by sequential incubation with specific primary antibodies (overnight at 4°C) and HRP-conjugated secondary antibodies (1 h at room temperature). β-tubulin served as an internal control. Protein bands were visualized using enhanced chemiluminescence substrate and quantitatively analyzed with ImageJ software.

### 2.14 Transmission electron microscope

Tissue fragments were promptly immersed in electron microscopy fixative and subjected to light-protected fixation at room temperature for 2 hours, followed by storage at 4°C. The fixed specimens were subsequently embedded, ultrathin sections were prepared, and ultrastructural observations were performed using transmission electron microscopy (TEM) with image acquisition.

### 2.15 Immunohistochemistry

Deparaffinized sections were subjected to heat-induced antigen retrieval using 10 mM citrate buffer. After 15-minute incubation with 3% hydrogen peroxide at room temperature, non-specific binding was blocked with bovine serum albumin. Sequential incubations with specific primary antibodies (4°C overnight) and HRP-conjugated secondary antibodies (room temperature for 1 hour) were performed, followed by chromogenic development using 3,3’-diaminobenzidine (DAB) substrate. Nuclei were counterstained with hematoxylin, differentiated with acidic ethanol solution, and blued in ammonia water. Sections were dehydrated through an ethanol gradient, cleared in xylene, and permanently mounted with neutral resin. Stained specimens underwent microscopic examination, with positive signals quantitatively analyzed using ImageJ software.

### 2.16 Statistical analysis

All statistical analyses were performed using SPSS 26.0 software. Continuous variables with normal distribution were expressed as mean ± standard deviation (SD), while non-normally distributed data were presented as median (interquartile range) [M (P25, P75)]. Between-group comparisons for normally distributed variables utilized independent samples t-test, whereas the wilcoxon rank-sum test was employed for non-normally distributed data. Multiple group comparisons were conducted using one-way ANOVA, with *post-hoc* analyses applying Least Significant Difference (LSD) method when homogeneity of variance was confirmed, and Tamhane’s T2 method when heterogeneity existed. A threshold of *P* < 0.05 indicated statistical significance.

## 3 Results

### 3.1 Phytochemical profiling of DHHL constituents

**Figure 1:**
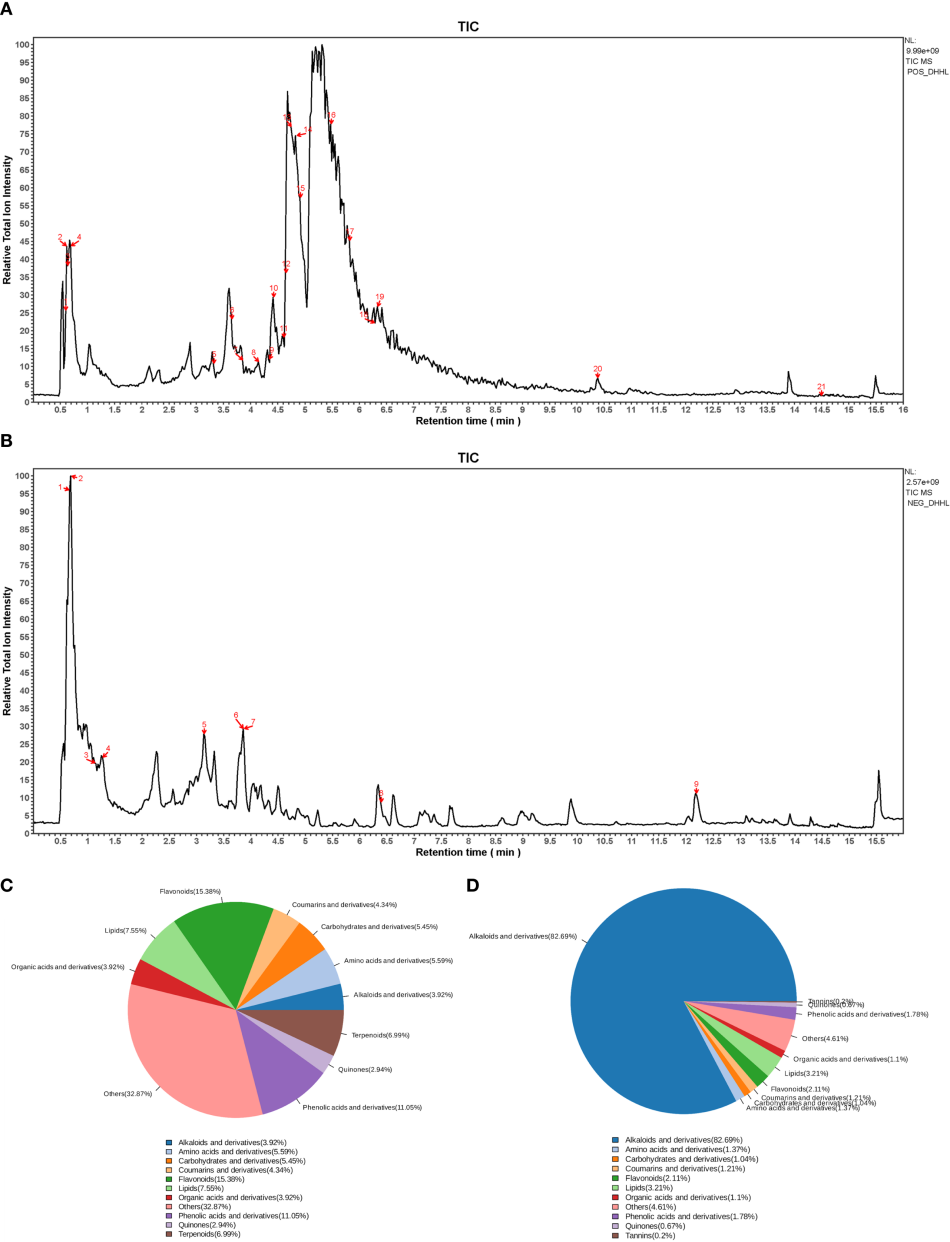
Identification of bioactive compounds in DHHL. **(A)** TIC in positive ion mode. **(B)** TIC in negative ion mode. **(C)** Distribution diagram of the quantity of component classification of DHHL. **(D)** Distribution diagram of component classification content of DHHL.

The chemical constituents of DHHL medicinal materials were identified and analyzed via UPLC-MS/MS, yielding the metabolite names, retention time (RT) in chromatography, mass-to-charge ratio (M/Z), molecular formula, ion detection mode (Mode), quality control (QC), and their botanical origins. Through non-targeted UPLC-MS/MS profiling coupled with database matching against MJBIOTCM (Majorbio’s specialized TCM metabolome database), we identified 777 distinct compounds as listed in Supplementary (**Supplementary Table S1**). From this comprehensive chemical inventory, we prioritized 10 herb-specific markers (e.g., emodin/berberine) based on two criteria: established bioactivity in Pharmacopoeia monographs, and documented relevance to metabolic regulation. Comprehensive analytical of these representative constituents are presented in (**Table 1**), providing a focused phytochemical signature of DHHL’s therapeutic potential. Subsequently, total ion chromatograms (TIC) were generated for all components under both positive and negative ion modes (**Figures 1A, B**). Based on subclass categorization from the identification results, pie charts illustrating the proportional distribution of compositional abundance (content percentage) and constituent quantity (subclass count) within different phytochemical categories were constructed (**Figures 1C, D**).

**Table 1:** Active pharmaceutical ingredient.

No.	RT(min)	M/z	Formula	Mode	QC	Compounds	Source
1	12.19	269.0456	C_15_H_10_O_5_	NEG	3467314.6	Emodin	Rhei Radix et Rhizoma
2	6.62	415.1038	C_21_H_20_O_9_	NEG	823271.17	Chrysophanol 8-O-beta-D-glucoside	Rhei Radix et Rhizoma
3	4.18	271.0601	C_15_H_10_O_5_	POS	1025974.7	Aloeemodin	Rhei Radix et Rhizoma
4	4.18	431.0986	C_21_H_20_O_10_	NEG	722172.14	Aloe-emodin-8-O-beta-D-glucopyranoside	Rhei Radix et Rhizoma
5	4.62	415.1039	C_21_H_20_O_9_	NEG	32822.755	Chrysophanol-1-O-b-D-glucoside	Rhei Radix et Rhizoma
6	7.68	445.1143	C_22_H_22_O_10_	NEG	178761.56	Physcion 8-O-beta-D-monoglucoside	Rhei Radix et Rhizoma
7	4.75	320.0918	C_19_H_14_NO_4+_	POS	352029843	Coptisine	Coptidis Rhizoma
8	9.88	283.0249	C_15_H_8_O_6_	NEG	1803546.8	Rheic acid	Coptidis Rhizoma
9	5.49	352.1538	C_21_H_22_NO_4+_	POS	488417711	Palmatine	Coptidis Rhizoma
10	4.84	336.1228	C_20_H_18_NO_4+_	POS	177154203	Epiberberine	Coptidis Rhizoma

### 3.2 HFD feeding induces obesity and glucose metabolism disorders in rats

**Figure 2:**
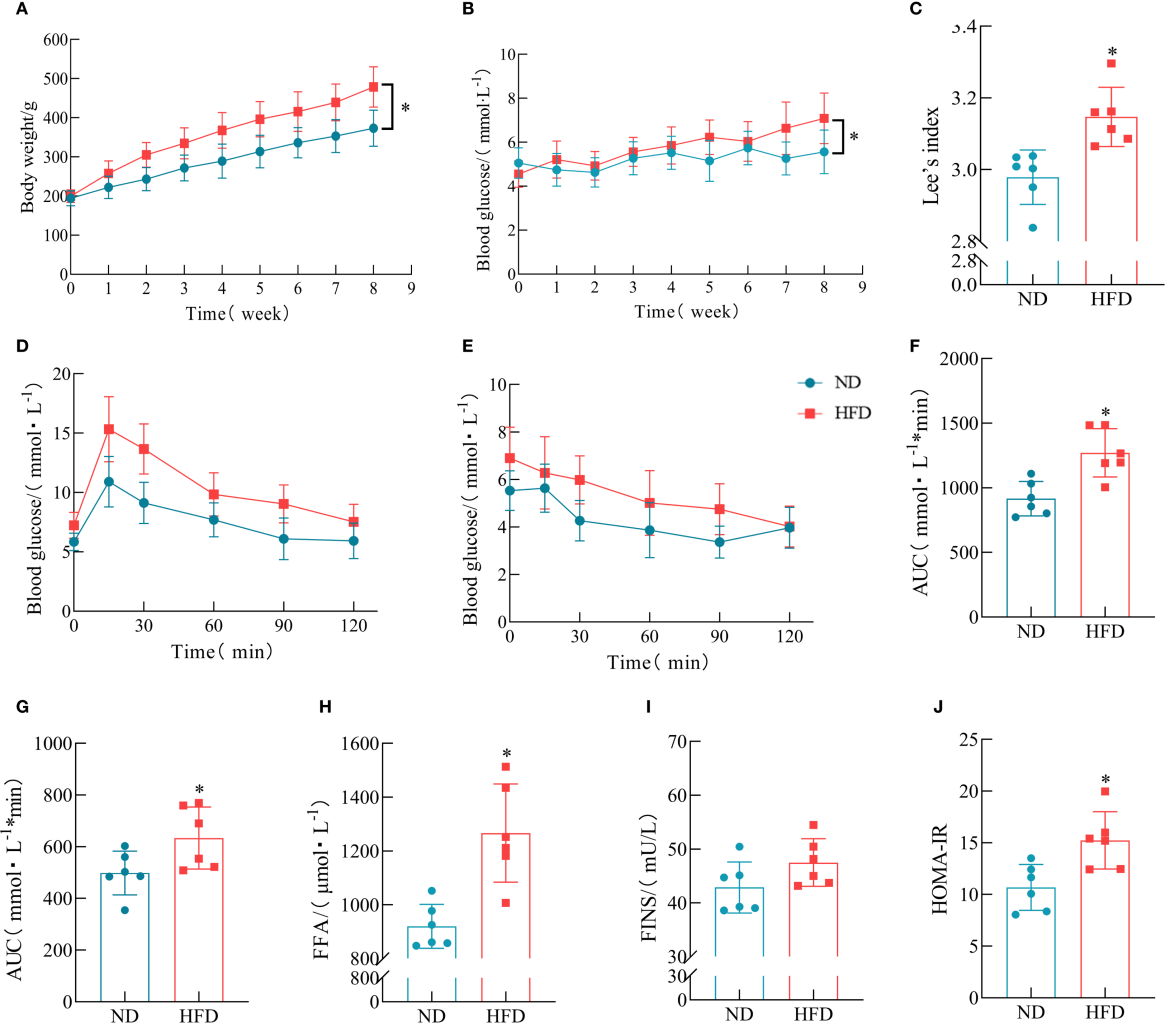
Metabolic consequences of HFD administration in the rat model. **(A)** Temporal dynamics of body weight across the 8-week intervention. **(B)** FBG levels from week 0 to week 8. **(C)** Lee’s index. (*n* = 6). **(D)** IPGTT. **(E)** ITT. **(F)** Area under the curve (AUC) of IPGTT blood glucose curve. (*n* = 6). **(G)** AUC of ITT blood glucose curve. (*n* = 6). **(H)** Serum FFA levels. (*n* = 6). **(I)** FINS levels. (*n* = 6). **(J)** HOMA-IR. (*n* = 6). (*P < 0.05, vs. the Control group).

An imbalance between energy intake and energy expenditure represents a fundamental pathological mechanism underlying obesity. Numerous studies have confirmed that HFD feeding significantly increases body weight, FBG, and other metabolic parameters, leading to systemic metabolic disorders (32). Our research also shows that after 8 weeks of HFD feeding, various physiological parameters were measured in both the ND group and the HFD group. Compared with rats in the ND group, those in the HFD group exhibited significantly increased body weight and FBG levels (**Figures 2A, B**). Further analysis using Lee’s index showed that the HFD group had a markedly higher Lee’s index than the ND group (**Figure 2C**). The results of the ITT demonstrated that the HFD group had severely impaired glucose handling capacity (**Figures 2D–G**). Moreover, rats fed with HFD demonstrated considerable elevation in serum concentrations of FFA and FINS when compared with their ND counterparts (**Figures 2H, I**). Concurrently, the HOMA-IR was substantially heightened in the HFD cohort (**Figure 2J**). These findings indicate that HFD feeding induces obesity and elevated FBG in rats.

### 3.3 HFD feeding promotes lipid accumulation in rats

**Figure 3:**
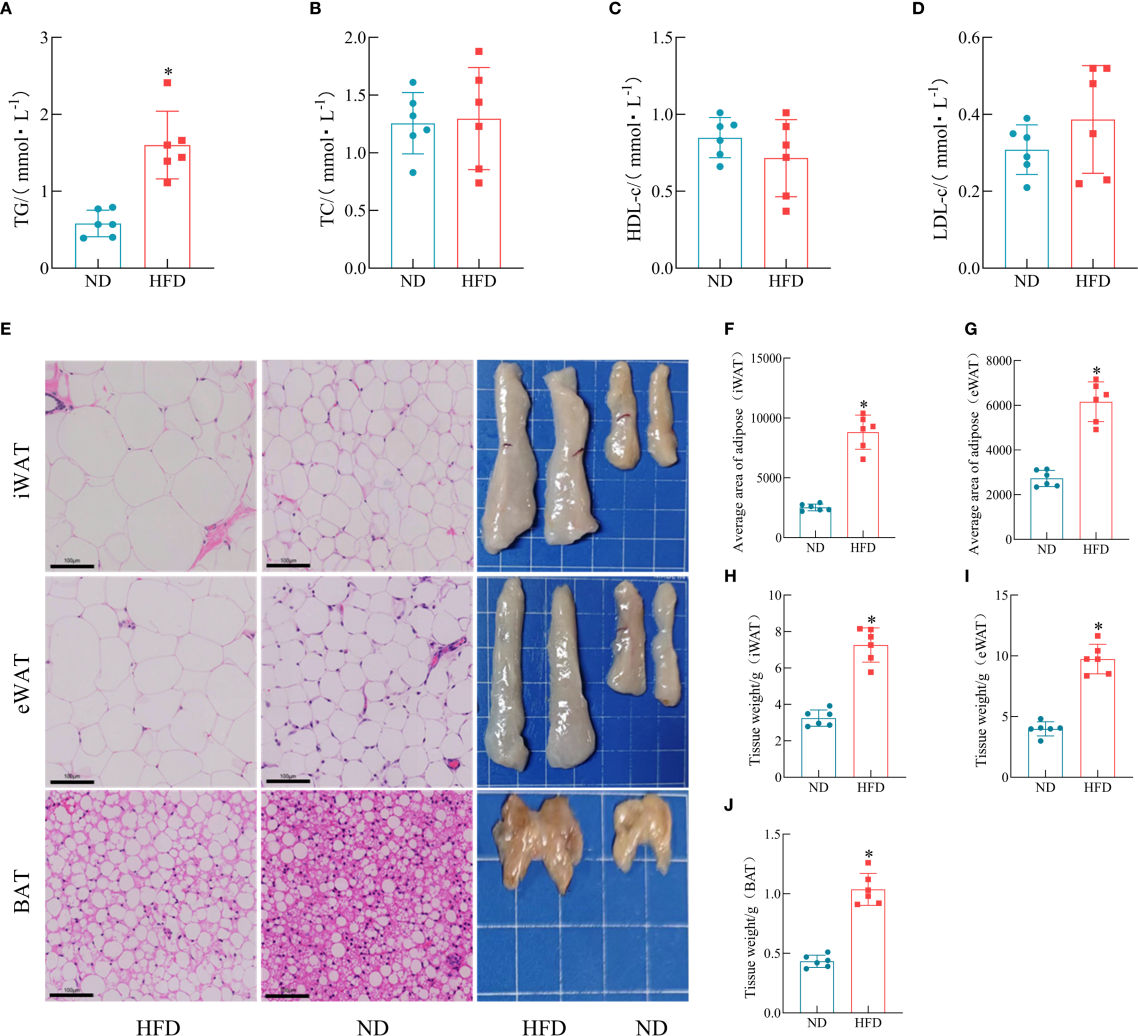
Effects of HFD on lipid accumulation in rats. **(A–D)** Serum levels of TG, TC, HDL-c, and LDL-c. (*n* = 6). **(E)** Representative anatomical images of iWAT, eWAT, and BAT, and corresponding H&E-stained sections. **(F–G)** Average adipocyte size in iWAT and eWAT. (*n* = 6). **(H–J)** Weights of iWAT, eWAT, and BAT. (*n* = 6). (*P < 0.05, vs. the Control group).

To further assess the impact of HFD on lipid metabolism, serum biochemical analysis was performed. The results showed that the TG levels were significantly elevated in HFD rats, whereas TC, HDL-c, with LDL-c profiles exhibiting statistical equivalence between the rodent populations subjected to either HFD or ND interventions (**Figures 3A–D**). As shown by the gross morphology of adipose tissues, the volumes of iWAT, epididymal white adipose tissue (eWAT), and BAT were markedly increased in HFD rats. H&E staining revealed that the adipocyte size in HFD rats was significantly larger than that in the ND group (**Figure 3E**). The extended mathematical analysis established that participants exposed to HFD manifested substantially higher values for both adipose tissue weight and adipocyte area relative to the ND group (**Figures 3F–J**).

### 3.4 DHHL improves body weight and blood glucose levels in obese rats

**Figure 4:**
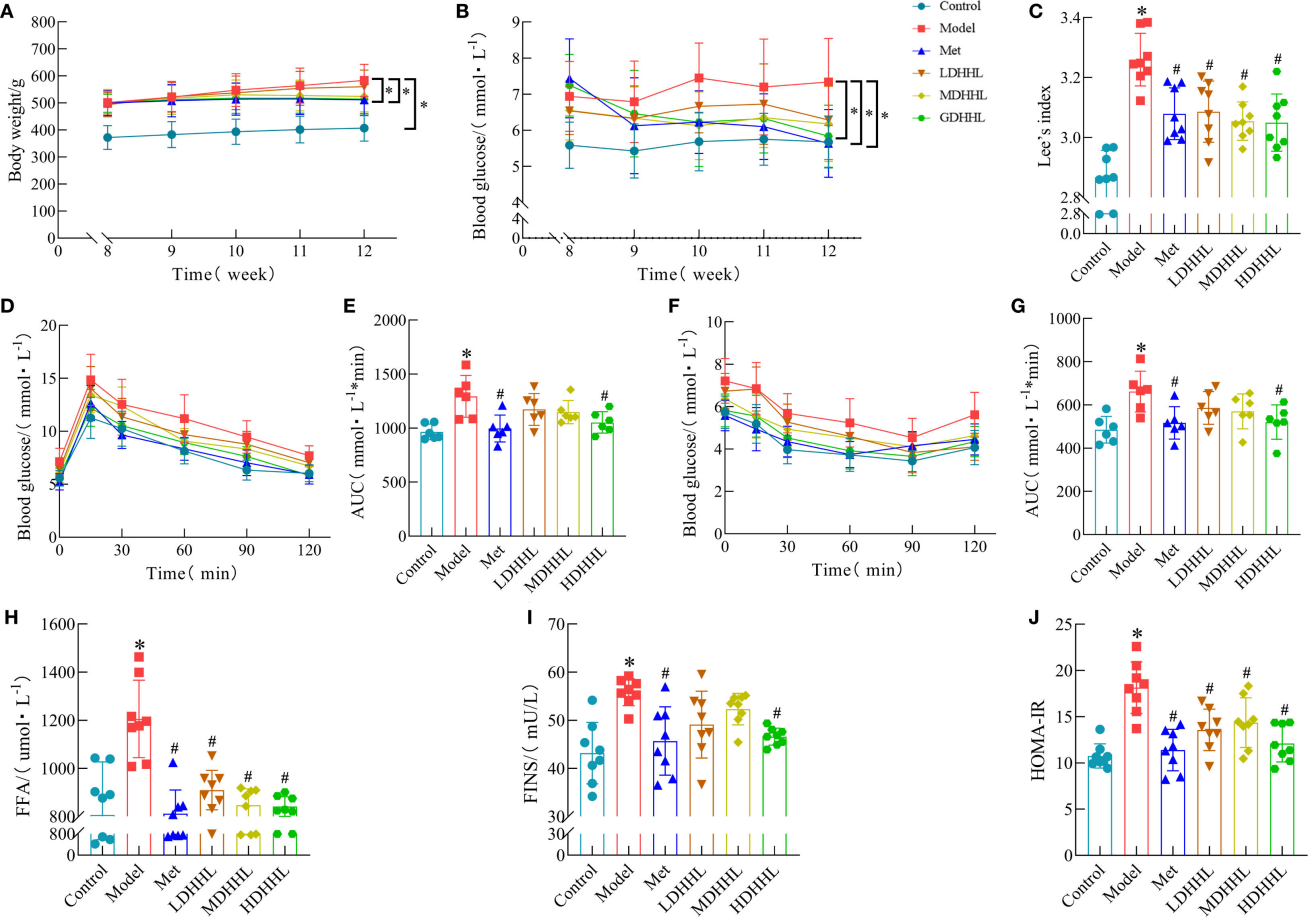
Physiological responses to DHHL administration in obesity-induced rats. **(A)** Chronological body mass documentation from weeks 8–12. **(B)** FBG levels during weeks 8–12. **(C)** Lee’s index. (*n* = 8). **(D)** IPGTT. **(E)** Quantitative analysis of IPGTT response using AUC methodology. (*n* = 6). **(F)** ITT. **(G)** AUC determination of glycemic fluctuations during ITT. (*n* = 6). **(H)** Serum FFA levels. (*n* = 8). **(I)** FINS levels. (*n* = 8). **(J)** HOMA-IR. (*n* = 8). (*P < 0.05, vs. the Control group; ^#^ *P* < 0.05, vs. the Model group).

Except for the ND group, all other groups continued to receive HFD feeding. Metformin was used as a positive control drug, and different doses of DHHL decoction were administered to the DHHL treatment groups. After 4 weeks of treatment, relevant indicators were measured. TCM offers inherent strengths in managing chronic metabolic disorders such as obesity. Existing studies have documented the significant efficacy of *Rhei Radix et Rhizoma* and *Coptidis Rhizoma* in ameliorating obesity, dyslipidemia, and glucose dysregulation (6, 13). Our prior research further confirms that the DHHL herb pair effectively improves metabolic parameters in genetically obese diabetic (db/db) mice (14). Rats in the model group showed continuous weight gain, which was significantly higher than that in the control group, whereas treatment with metformin and HDHHL significantly suppressed HFD-induced weight gain (**Figure 4A**). The assessment of Lee’s index revealed a substantial increase in rats assigned to the model group; however, intervention with either metformin or DHHL effectively diminished this parameter, indicative of obesity improvement (**Figure 4C**). Rats in the model cohort demonstrated notably increased FBG measurements; however, intervention with DHHL therapy resulted in a considerable reduction of these elevated glucose levels (**Figure 4B**). The results of IPGTT and ITT assessments indicated substantial compromise in glucose utilization capacity and insulin sensitivity parameters among subjects in the model group; these impairments were notably ameliorated following DHHL administration, indicating improvements in glucose and insulin tolerance (**Figures 4D–G**). Analysis of serum samples demonstrated that model group subjects manifested substantially higher levels of FFA, FINS, and HOMA-IR compared to the control cohort; subsequent DHHL therapy effectively reduced these elevated biochemical markers (**Figures 4H–J**).

### 3.5 DHHL alleviates lipid accumulation in obese rats

**Figure 5:**
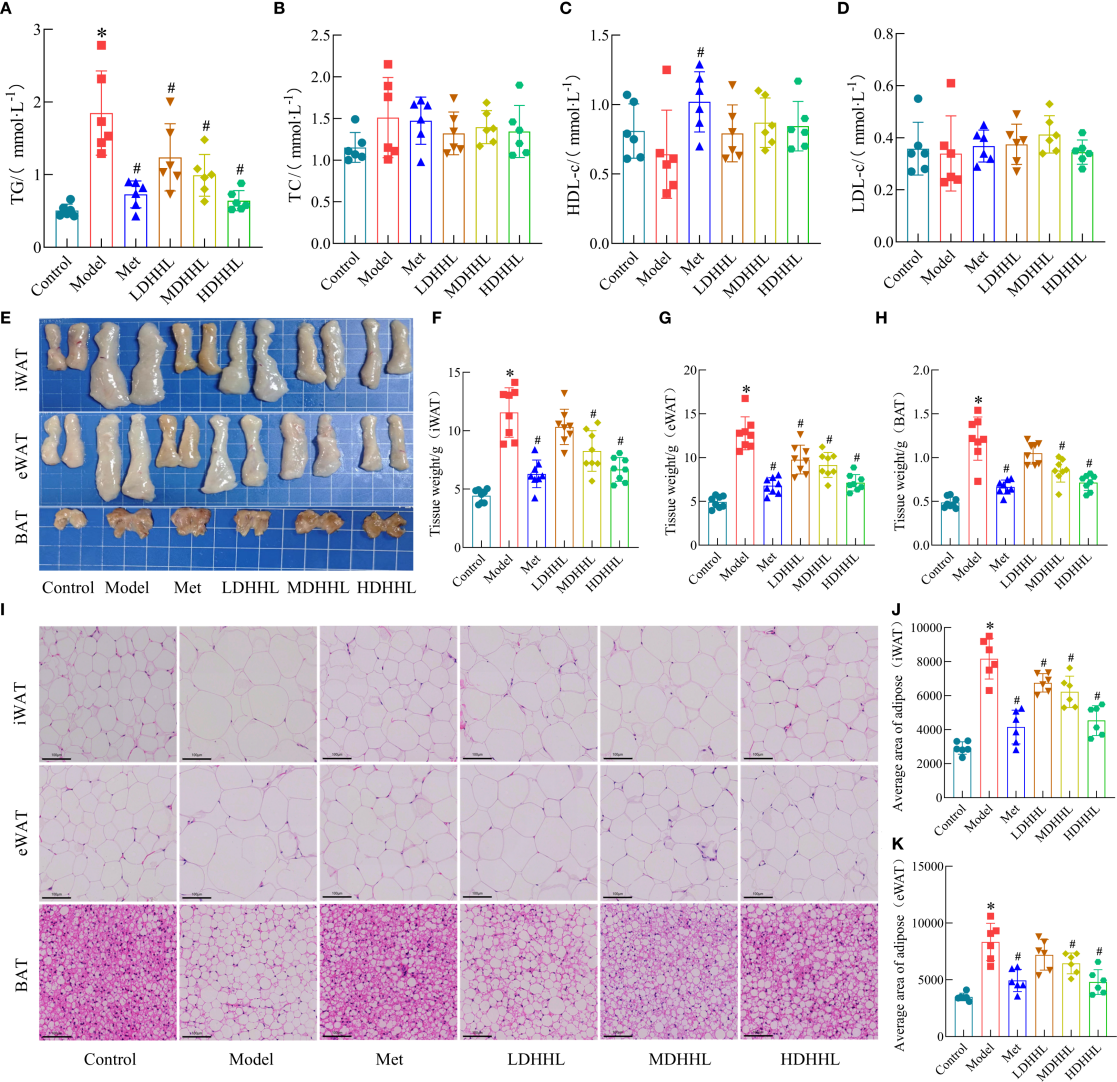
Effects of DHHL on lipid accumulation in obese rats. **(A–D)** TG, TC, HDL-c, and LDL-c concentrations in serum. (*n* = 6). **(E)** Gross anatomical images of iWAT, eWAT, and BAT. **(F–H)** Weights of iWAT, eWAT, and BAT. (*n* = 8). **(I)** Characteristic H&E-stained tissue sections from iWAT, eWAT, and BAT. **(J–K)** Average adipocyte size in iWAT and eWAT. (*n* = 6). (*P < 0.05, vs. the Control group; ^#^ *P* < 0.05, vs. the Model group).

As shown in the figure below, the TG content in the model group was significantly higher than that in the control group, whereas DHHL administration significantly reduced serum TG levels in rats (**Figure 5A**). However, only slight differences were observed among groups regarding serum levels of TC, LDL-c, and HDL-c (**Figures 5B–D**). Gross morphology of adipose tissues revealed that iWAT, eWAT, and the model group displayed substantially enlarged and heavier BAT relative to the control group, with different dosages of DHHL treatment producing variable degrees of diminution in both the size and weight of adipose tissue (**Figures 5E–H**). H&E staining was used to analyze adipose tissues from each group. Data indicated that adipocytes were substantially larger in the model group, and treatment with various doses of DHHL resulted in graduated diminution of adipocyte dimensions (**Figures 5I–K**). In summary, DHHL effectively improved body fat composition in obese rats, and its therapeutic effect exhibited a dose-dependent trend.

### 3.6 The therapeutic effect of DHHL on obese rats is closely associated with enhanced thermogenesis and WAT browning

**Figure 6:**
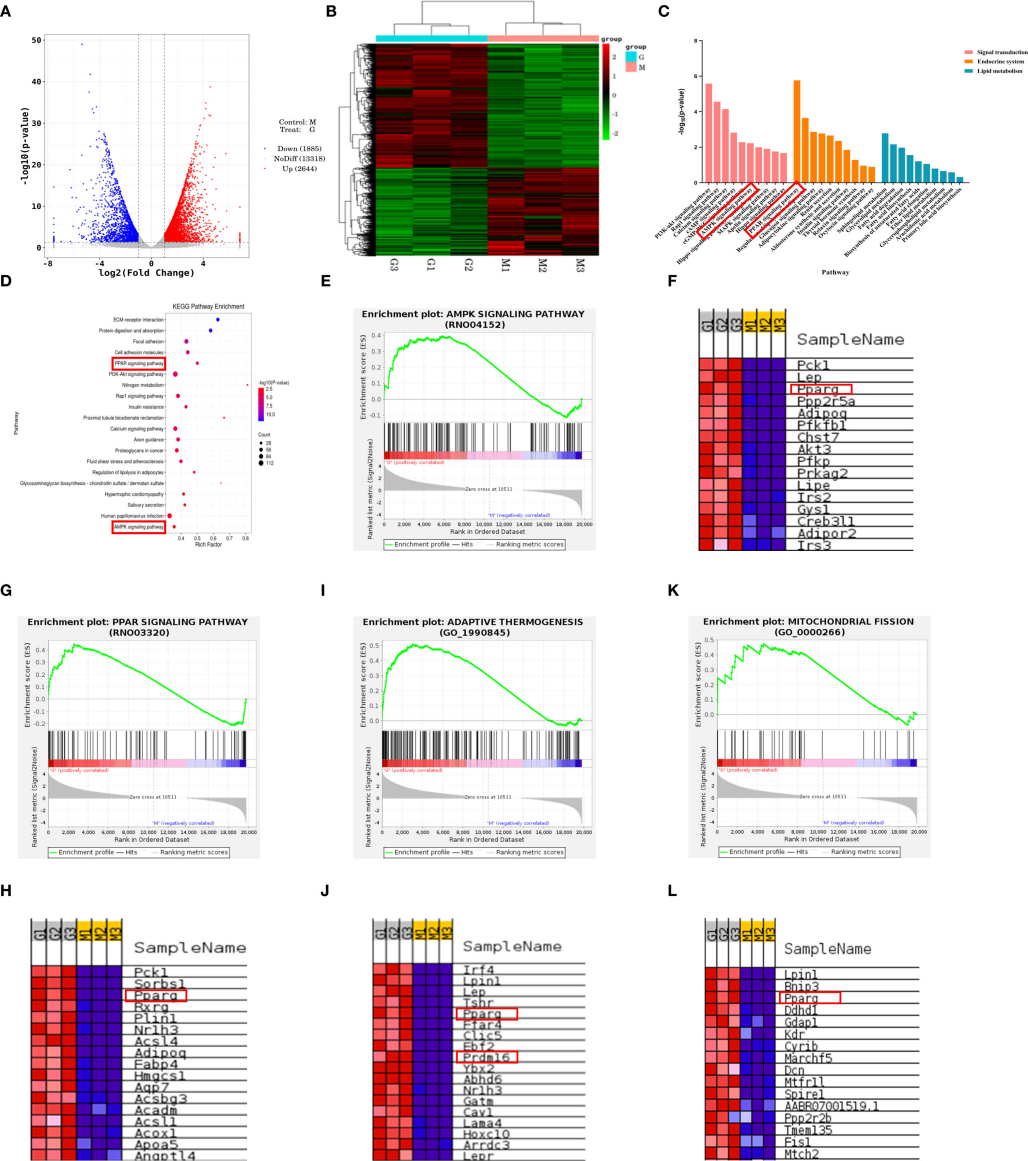
Transcriptomic sequencing analysis of iWAT in obese rats and DHHL-treated rats. **(A)** Volcano plot. **(B)** Hierarchical clustering heatmap. **(C)** KEGG classification bar chart. **(D)** KEGG pathway enrichment bubble plot. **(E-L)** GSEA enrichment plots and associated differential expression heatmaps for AMPK signaling, PPAR signaling, adaptive thermogenesis, and mitochondrial fission. (M denotes Model group rats; G indicates HDHHL-treated rats. Red: upregulated; Blue: downregulated).

The aforementioned experiments have confirmed that DHHL can significantly improve body weight and glucose metabolism disorders in obese rats, exhibiting a dose-dependent therapeutic effect. To further elucidate the mechanism by which DHHL effectively alleviates obesity in rats, we performed transcriptomic sequencing analysis on iWAT from rats in the Model group and the HDHHL group. Visualization via volcano plot demonstrated a considerable quantity of genes with differential expression patterns when comparing the Model and HDHHL cohorts (**Figure 6A**). In alignment with these findings, hierarchical clustering visualization via heatmap revealed pronounced distinctions in iWAT transcriptomic signatures between the compared groups (**Figure 6B**). To investigate the potential biological pathways involved, we constructed KEGG enrichment bar charts covering signaling pathways, the endocrine system, and lipid metabolism (**Figure 6C**), as well as KEGG enrichment bubble plots (**Figure 6D**). Both figures showed significant enrichment in pathways related to WAT browning, notably the AMPK and PPAR signaling cascades. Gene set enrichment analysis (GSEA) revealed that the gene sets of AMPK signaling pathway (**Figure 6E**), PPARγ-associated pathways (**Figure 6G**), adaptive thermogenesis (**Figure 6I**), and mitochondrial fission (**Figure 6K**) exhibited significant upregulation in the HDHHL group compared with the Model group. Furthermore, differential heatmap analysis visually confirmed that key regulators of adaptive thermogenesis and white adipose tissue browning, including PPARγ and PRDM16, exhibited significantly reduced expression in the Model group with concurrent marked elevation in the HDHHL intervention group (**Figure 6J**).

### 3.7 DHHL promotes WAT browning in obese rats

**Figure 7:**
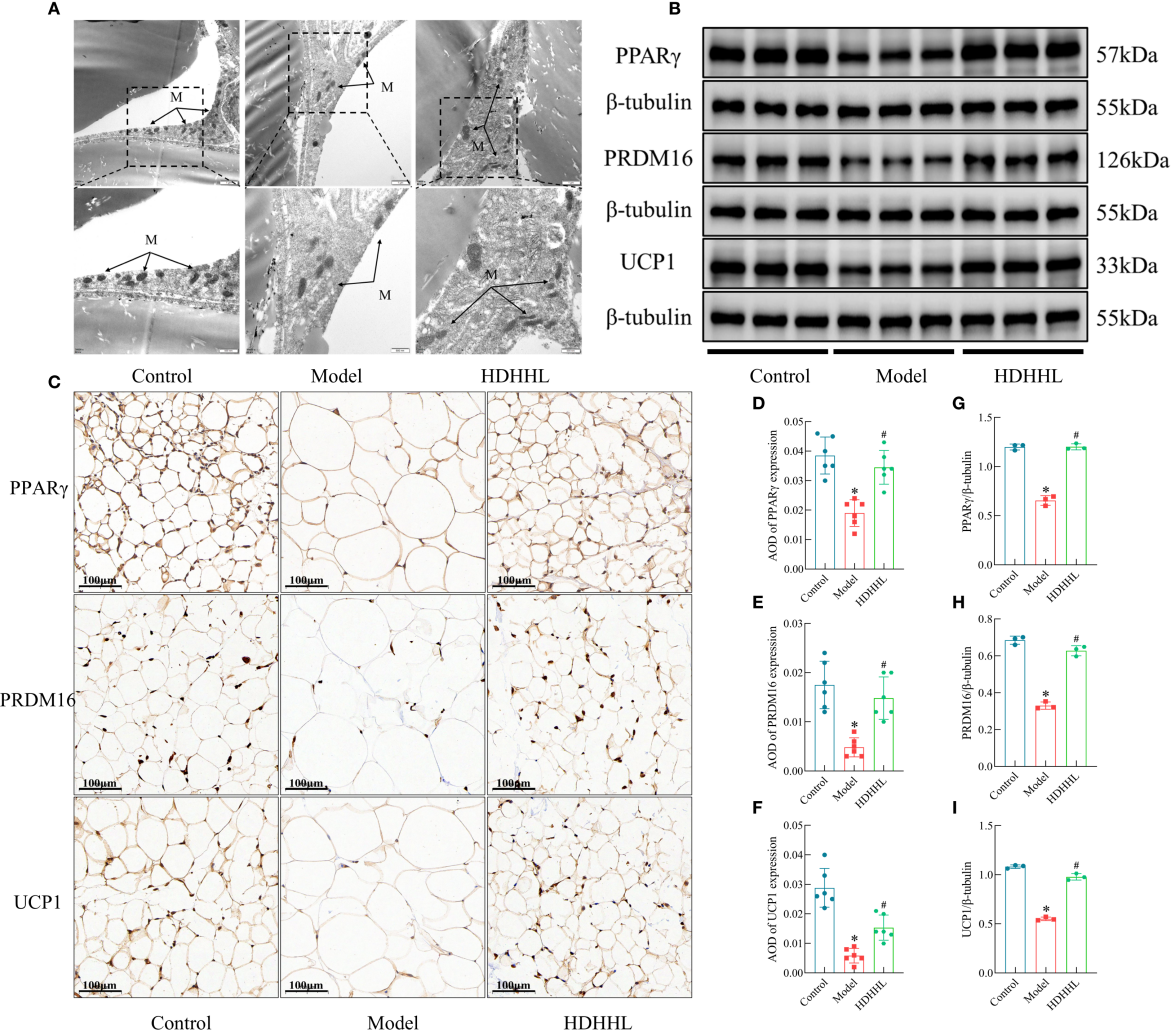
Effects of DHHL on WAT browning in obese rats. **(A)** Transmission electron microscopy images of iWAT. "M" stands for mitochondria. **(B, C)** Western blot bands and immunohistochemical staining images for PPARγ, PRDM16, and UCP1. **(D–I)** Quantitative analysis of PPARγ, PRDM16, and UCP1 protein expression from Western blotting (*n* = 3) and IHC (*n* = 6). (*P < 0.05, vs. the Control group; ^#^ *P* < 0.05, vs. the Model group).

The above transcriptomic analysis results indicate that DHHL may exert a regulatory effect on genes associated with WAT browning. Hallmark features of WAT browning include increased mitochondrial abundance within adipocytes and elevated expression of thermogenesis-related proteins such as PPARγ, PRDM16, and UCP1. Furthermore, several studies have confirmed that rhubarb and coptis can promote WAT browning. Berberine, the primary active constituent of coptis, enhances WAT browning in both 3T3-L1 murine preadipocyte cells and obese mice (13), thereby ameliorating obesity. Dietary supplementation with rhubarb similarly induces WAT browning, augmenting thermogenesis and energy expenditure to counteract metabolic syndrome (33). Therefore, this study further examined the regulatory role of DHHL in promoting WAT browning in rats. Transmission electron microscopy revealed that, in contrast to the Control group, a substantial diminution in mitochondrial population was observed within the Model group; following DHHL treatment, mitochondrial abundance was partially restored (**Figure 7A**). Western blotting (WB) and immunohistochemistry (IHC) analyses showed that thermogenesis-related proteins were highly expressed in the adipose tissue of rats in the Control group. The HFD-induced obese rodent cohort, however, displayed substantially reduced levels of these proteins when iWAT tissue was examined. Notably, DHHL administration markedly reversed the HFD-induced suppression of thermogenesis-related protein expression in iWAT (**Figures 7B–I**). These findings collectively suggest that DHHL promotes WAT browning in obese rats.

### 3.8 DHHL promotes WAT browning in obese rats via activation of the AMPK/SIRT1/PGC-1α pathway

**Figure 8:**
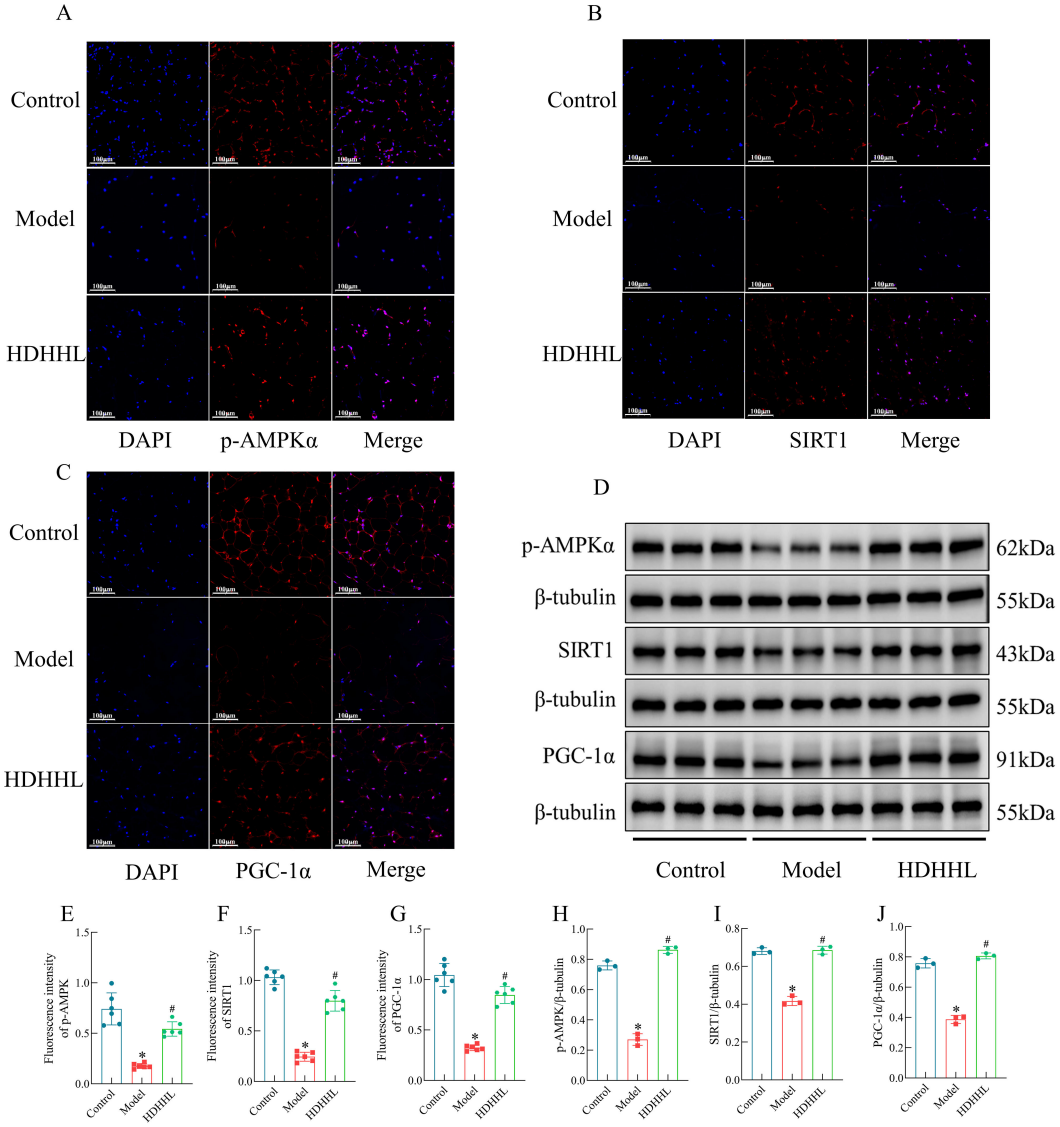
Effects of DHHL on the expression of proteins in the AMPK/SIRT1/PGC-1α pathway. **(A–D)** FIHC images and Western blot bands for p-AMPKα, SIRT1, and PGC-1α. **(E–J)** Quantitative analysis of p-AMPKα, SIRT1, and PGC-1α protein expression by FIHC (*n* = 6) and WB (*n* = 3). (*P < 0.05, vs. the Control group; ^#^ *P* < 0.05, vs. the Model group).

Earlier investigations have revealed the essential function of the AMPK/SIRT1/PGC-1α pathway in modulating the browning process of white adipose tissue (28, 29). Consistently, transcriptomic analyses in the present study also indicated that the therapeutic effect of DHHL on obese rats is closely associated with activation of the AMPK pathway. We sought additional confirmation regarding DHHL’s obesity-alleviating effects through WAT browning enhancement via the AMPK/SIRT1/PGC-1α pathway by evaluating crucial protein expressions within iWAT across different groups employing fluorescence immunohistochemistry (FIHC) (**Figures 8A–C**) and WB (**Figure 8D**), followed by quantitative statistical analysis presented as bar graphs (**Figures 8E–J**). Examination of iWAT revealed that subjects in the Model group displayed considerably reduced levels of phosphorylated AMPKα (p-AMPKα), SIRT1, and PGC-1α expression compared to their Control counterparts. Notably, DHHL treatment markedly restored the expression of these proteins. These findings suggest that DHHL may promote iWAT browning by activating the AMPK/SIRT1/PGC-1α signaling pathway, which could be a key mechanism underlying its anti-obesity effects.

## 4 Discussion

The past few years have witnessed a continual surge in obesity prevalence, largely driven by altered eating patterns and steadily diminishing levels of physical exercise (2). Alongside this trend, the burden of obesity-related comorbidities has also been escalating annually. Carrying excess body weight substantially increases one’s susceptibility to cardiovascular diseases, diabetes, renal disorders, and various malignancies (34). Multiple complex determinants contribute to obesity’s etiology, whereby its pathogenic progression stems predominantly from long-standing discrepancies between energetic input and output, resulting in an energy surplus within the body. In response to this overnutrition, adipose tissue undergoes hypertrophy and hyperplasia to store excess energy, ultimately contributing to overweight and obesity if sustained over time (35). Congruent with the described mechanisms, we observed that rats maintained on a HFD developed substantial weight increases accompanied by heightened Lee’s index parameters. Comparative analysis revealed elevated serum TG and FFA levels in relation to subjects in the ND group. Furthermore, HFD-fed rats displayed significantly higher FBG, FINS, and HOMA-IR, indicating the presence of glucose metabolism disorder. The HFD-fed subjects exhibited enlarged adipocyte structures and increased fat mass when contrasted with the ND group. Given the growing severity of obesity and the inherent limitations of existing therapeutic interventions, there is an urgent need to explore more rational and feasible strategies for prevention and treatment.

In this study, we selected Da Huang and Huang Lian—two classical bitter-cold herbs from the traditional Chinese formula Da Huang Huang Lian Xie Xin Tang—for the treatment of obesity. Both herbs are characterized by their bitter and cold properties. From the perspective of modern medicine, obesity is closely associated with an imbalance in energy metabolism, involving enhanced lipogenesis and reduced lipolysis (3). Studies have demonstrated that *Rhei Radix et Rhizoma*, *Coptidis Rhizoma*, or their major bioactive constituents can effectively alleviate obesity and improve disturbances in glucose and lipid metabolism (5, 12). Our previous research has confirmed that DHHL can significantly reduce elevated body weight and blood glucose levels in db/db mice (14). Consistent with those findings, the present study also indicates that DHHL contains multiple active compounds capable of mitigating HFD-induced obesity in rats by lowering body weight, blood glucose, and lipid levels. Moreover, DHHL partially reversed the HFD-induced increases in adipose tissue mass and lipid droplet area. Nevertheless, the mechanisms responsible for these observed effects await comprehensive clarification.

To further investigate the mechanisms by which DHHL ameliorates HFD-induced obesity in rats, we performed transcriptomic sequencing analysis on the iWAT of rats from the Model group and the HDHHL group, aiming to uncover the potential pathways and molecular targets through which the herbal formulation exerts its therapeutic effects. Transcriptomics is a discipline that investigates gene transcription and transcriptional regulatory patterns at a genome-wide level, focusing on the differential expression of genes at the RNA level (15). Sequencing analysis revealed significant differences in gene expression within the iWAT between the Model group and the HDHHL group. GSEA demonstrated that HDHHL significantly upregulated the expression of thermogenesis-related gene sets, including PPARγ and PRDM16, whereas the expression of thermogenic genes was suppressed in the Model group. KEGG pathway enrichment analysis indicated that the therapeutic effects of DHHL on obesity were closely associated with the promotion of WAT browning, and that the AMPK and PPAR signaling pathways may serve as key mechanisms by which DHHL exerts its anti-obesity effects.

Recent studies have shown that WAT browning activates thermogenic programs that facilitate the dissipation of excess energy as heat. This mechanism of non-exercise-induced energy expenditure has opened new avenues for the metabolic treatment of obesity (36). Mitochondria, as central regulators of cellular energy metabolism, reflect the energy consumption status of cells. Following the browning of WAT, mitochondrial biogenesis is enhanced, the number of mitochondria increases, and the expression of thermogenesis-related proteins such as UCP1, PRDM16, and PPARγ is significantly upregulated, thereby promoting energy expenditure (37). In this study, the ultrastructural features of iWAT cells in each group of rats were examined using transmission electron microscopy. Examination of iWAT cellular structures indicated lower mitochondrial density in the Model group, while administration of DHHL partially restored the mitochondrial depletion. Quantitative analysis of thermogenesis-related markers (UCP1, PRDM16, and PPARγ in iWAT was performed using IHC and WB. The results indicated that a HFD markedly suppressed the expression of thermogenic proteins; however, DHHL treatment restored the expression balance of proteins associated with white adipose tissue browning, significantly reversing the inhibitory effects on these proteins.

As a key regulatory target in cellular metabolism of substances and energy, AMPK can promote white adipose tissue browning by activating the SIRT1/PGC-1α signaling pathway. Dietary kaempferol-induced browning of WAT has been shown to involve the AMPK/SIRT1/PGC-1α pathway, and the browning phenotype in kaempferol-treated cells was partially reversed by the use of AMPK inhibitors (28). Mulberry leaf flavonoid compounds induce PPARγ and UCP1 gene expression via the AMPK/SIRT1/PGC-1α signaling cascade, thereby promoting BAT activation and WAT browning, ultimately improving type 2 diabetes mellitus (38). Utilizing data derived from transcriptomic sequencing, we subsequently explored expression profiles of molecular components that participate in AMPK-dependent signaling. The expression levels of p-AMPKα, SIRT1, and PGC-1α in iWAT were assessed using FIHC and WB. Within the obese rat population, researchers noted considerable decreases in both AMPK phosphorylation levels and the expression of SIRT1 and PGC-1α, which function as downstream elements in the AMPK signaling network. Treatment with DHHL partially restored AMPK activity and the expression levels of downstream proteins. These findings suggest that activation of the AMPK/SIRT1/PGC-1α pathway may be a potential mechanism by which DHHL enhances WAT browning and consequently ameliorates obesity. However, this study did not further validate this mechanism at the cellular level. Moreover, the specificity of AMPK as a potential molecular target of DHHL requires validation through reverse pharmacological approaches such as gene silencing or the use of specific inhibitors (e.g., Compound C), which will constitute the next phase of our investigation into the therapeutic efficacy of DHHL in obesity management.

## 5 Conclusion

In conclusion, this study demonstrates that DHHL effectively ameliorates HFD-induced obesity and mitigates glucose-lipid metabolic disorders in rats. Through integrated transcriptomic sequencing and molecular biology validation, we revealed that DHHL activates the AMPK/SIRT1/PGC-1α signaling axis and promotes WAT browning, suggesting this pathway constitutes the potential therapeutic mechanism underlying DHHL’s anti-obesity effects. These findings provide experimental evidence and novel insights into TCM interventions for metabolic disorders including obesity.

## Data Availability

The datasets presented in this study can be found in online repositories. The names of the repository/repositories and accession number(s) can be found in the article/**Supplementary Material**.
