# Supplementary figures and images for: Transcriptomic elucidation of Dahuang-Huanglian in promoting white adipose browning in high-fat diet-induced obese rats
Source: Front Endocrinol (Lausanne). 2025 Oct 8;16:1652703. doi: 10.3389/fendo.2025.1652703 (PMC12540174; doi:10.3389/fendo.2025.1652703)

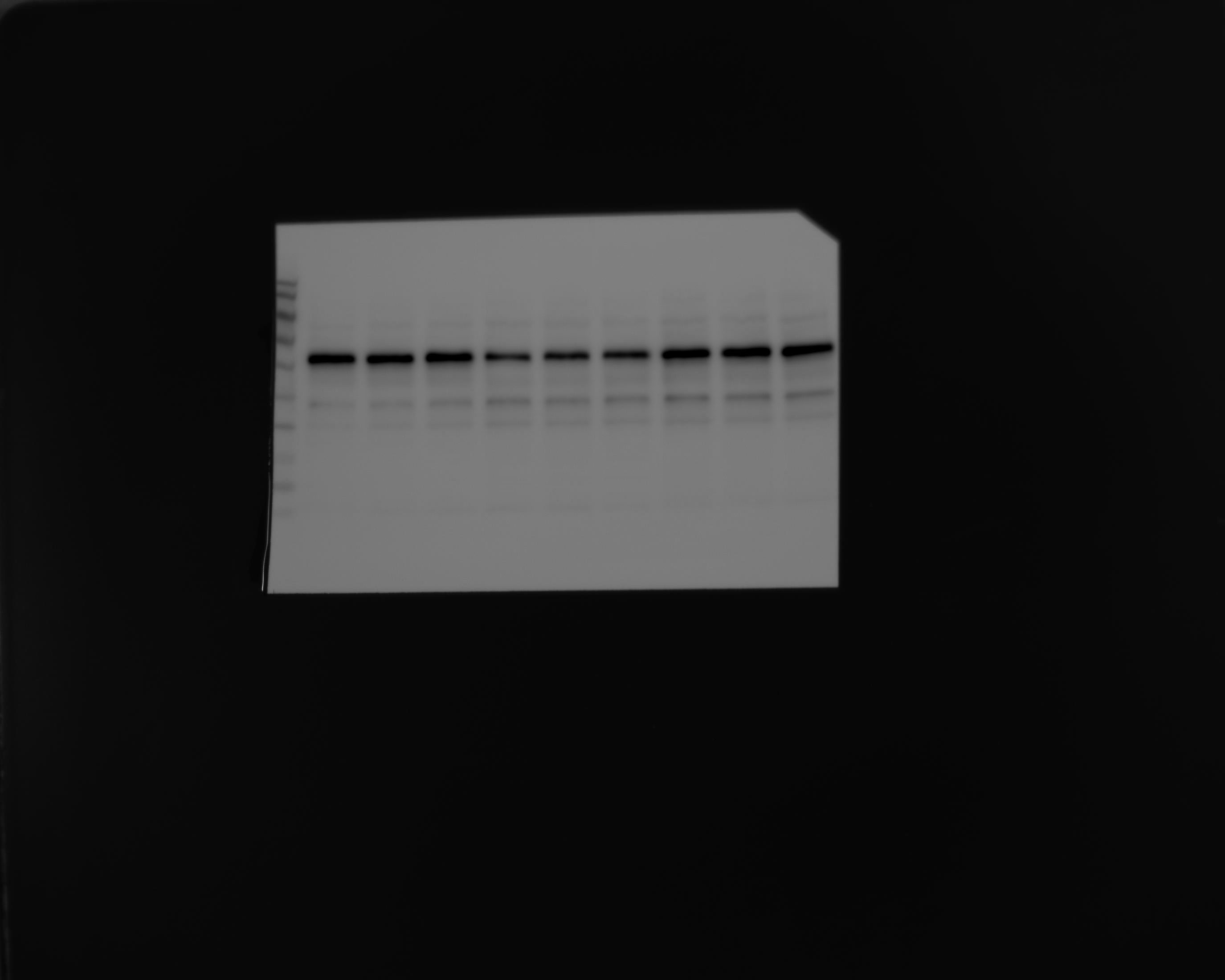

Supplement: Supplementary file 1 [file Image1.jpeg]

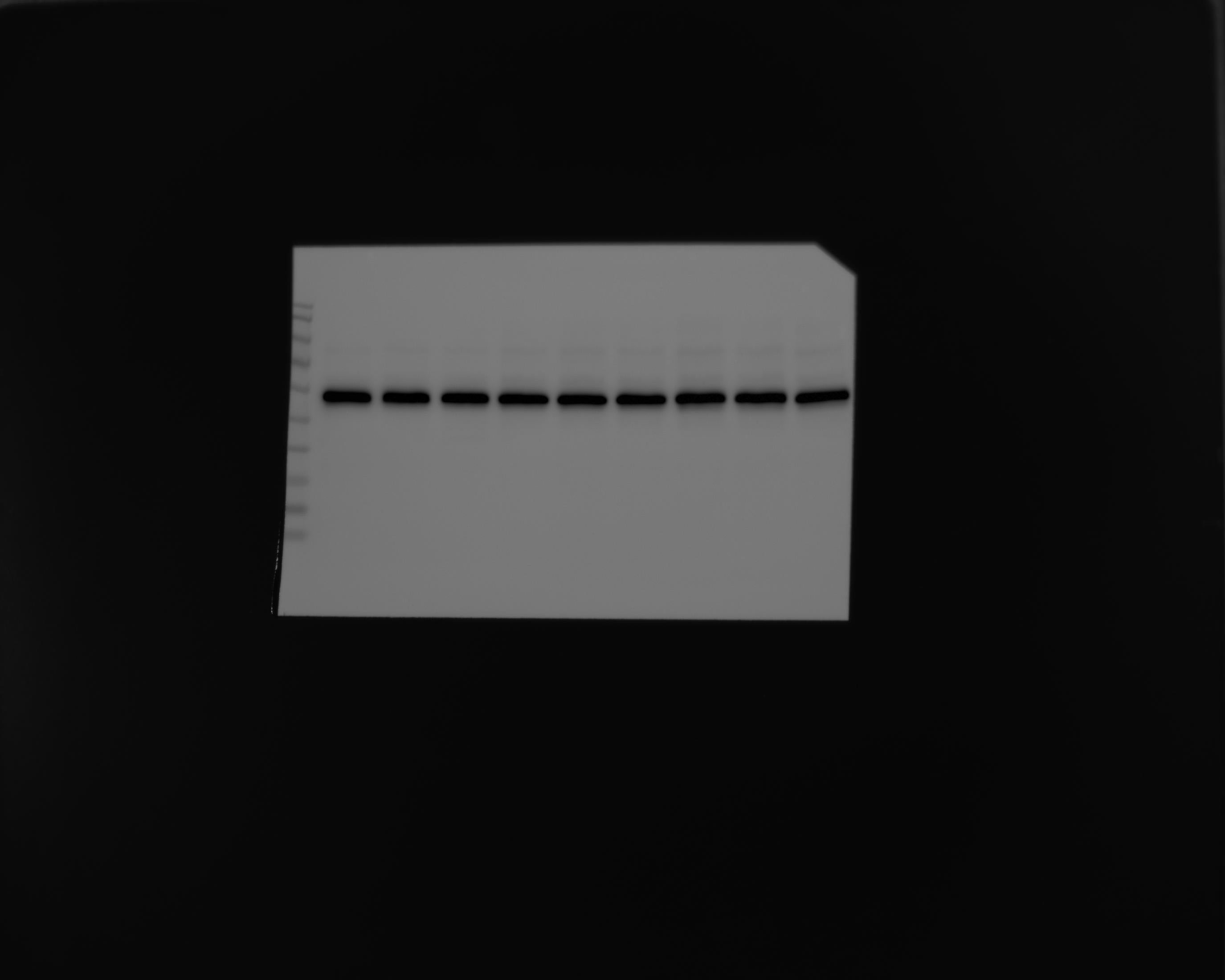

Supplement: Supplementary file 2 [file Image2.jpeg]

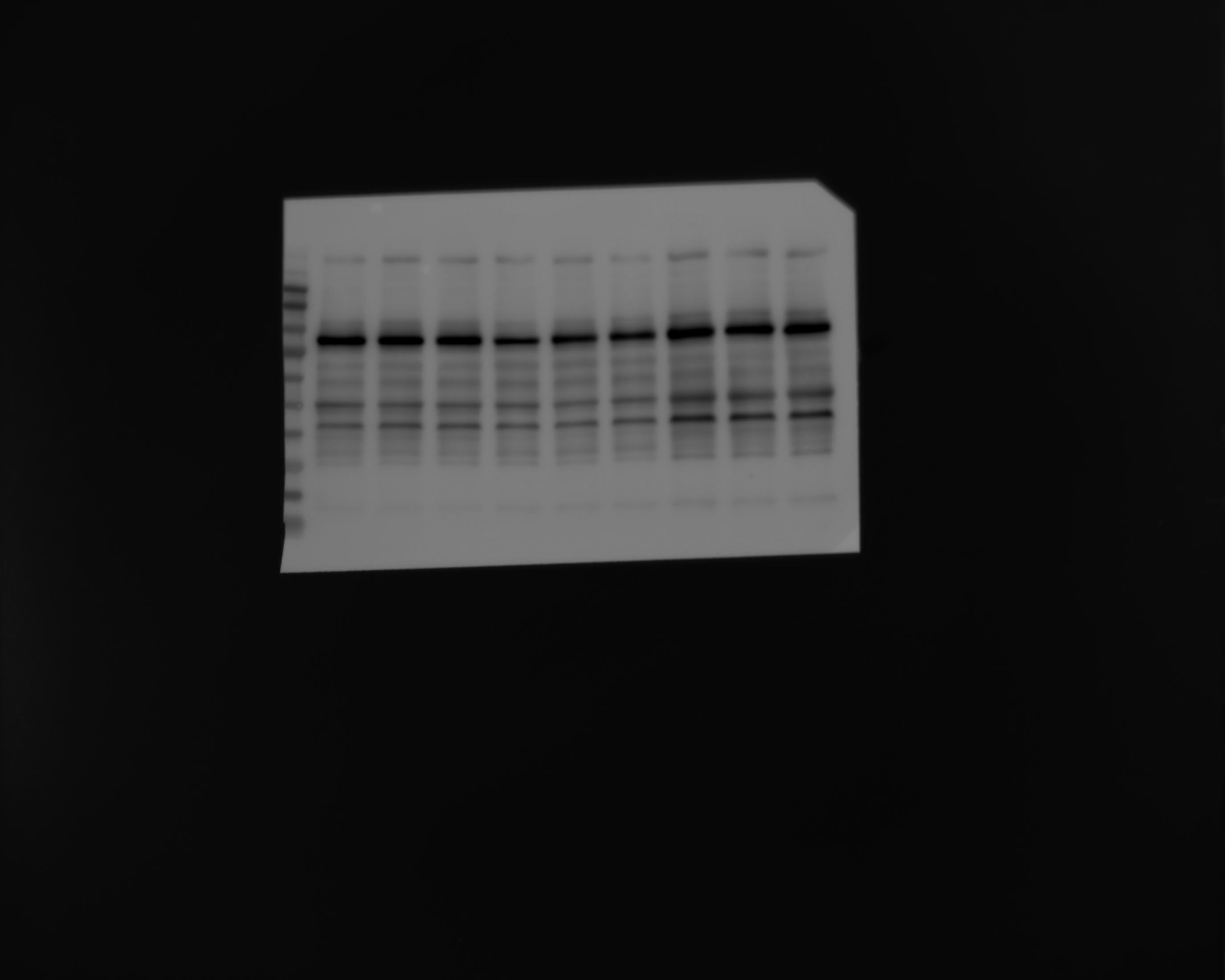

Supplement: Supplementary file 3 [file Image3.jpeg]

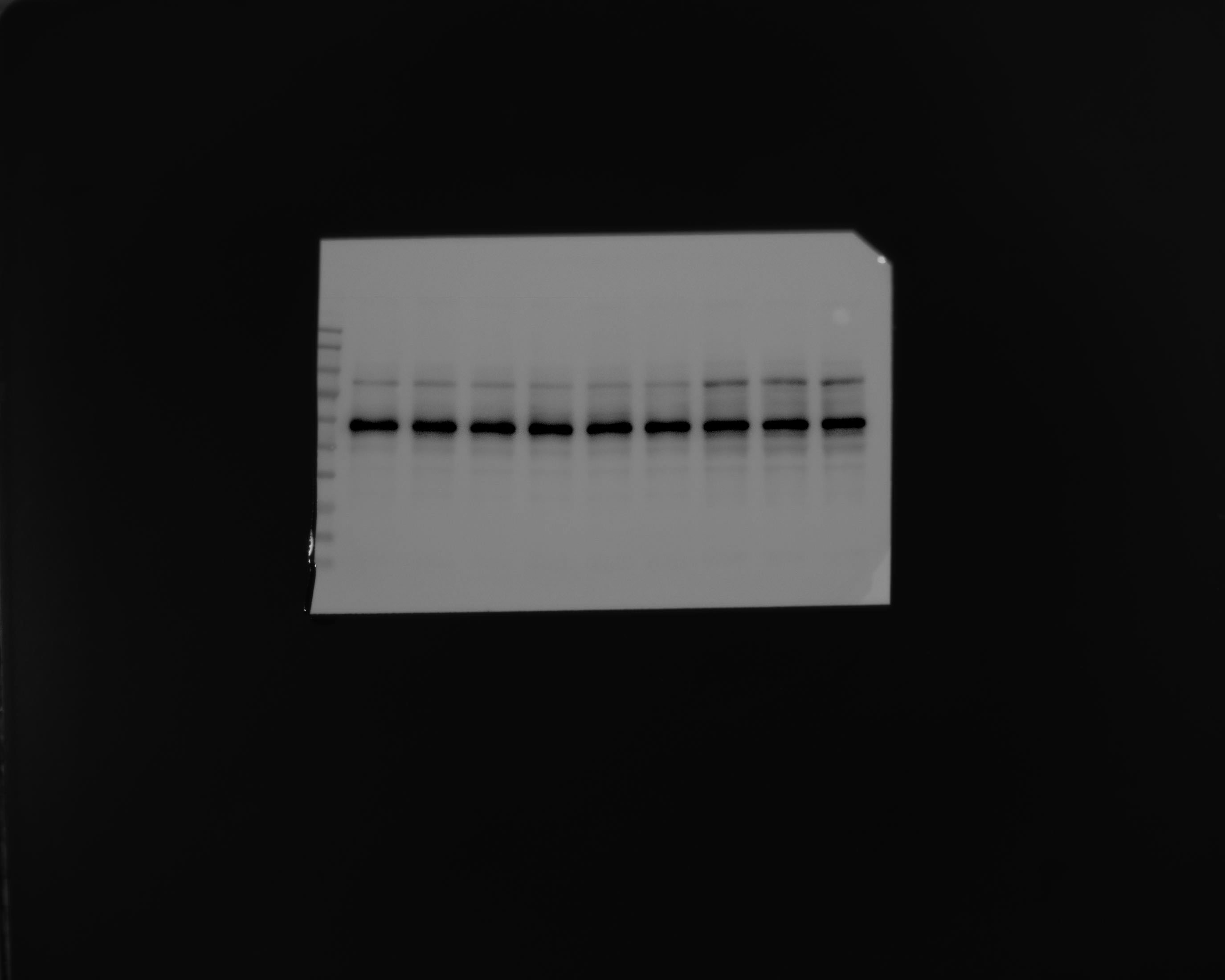

Supplement: Supplementary file 4 [file Image4.jpeg]

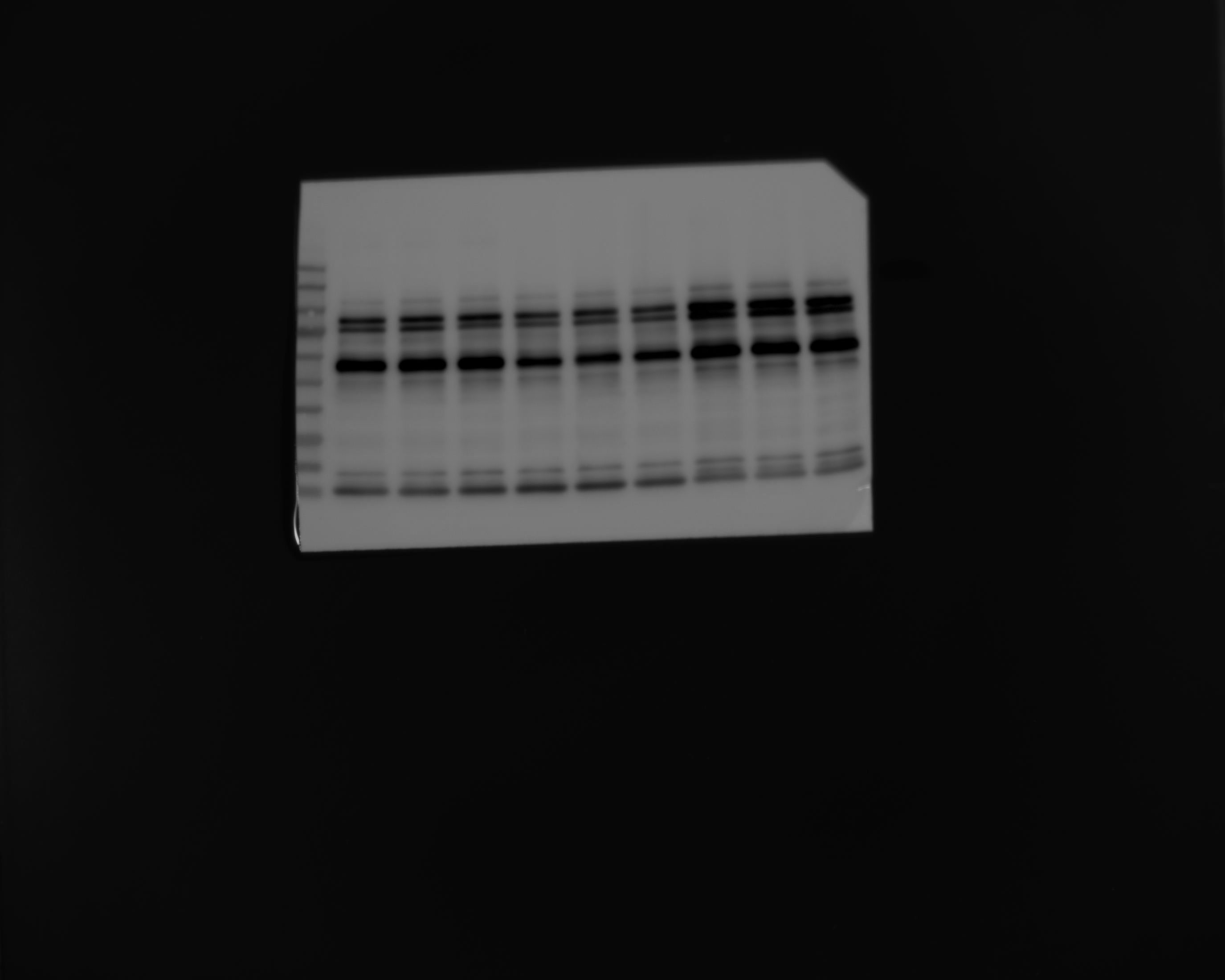

Supplement: Supplementary file 5 [file Image5.jpeg]

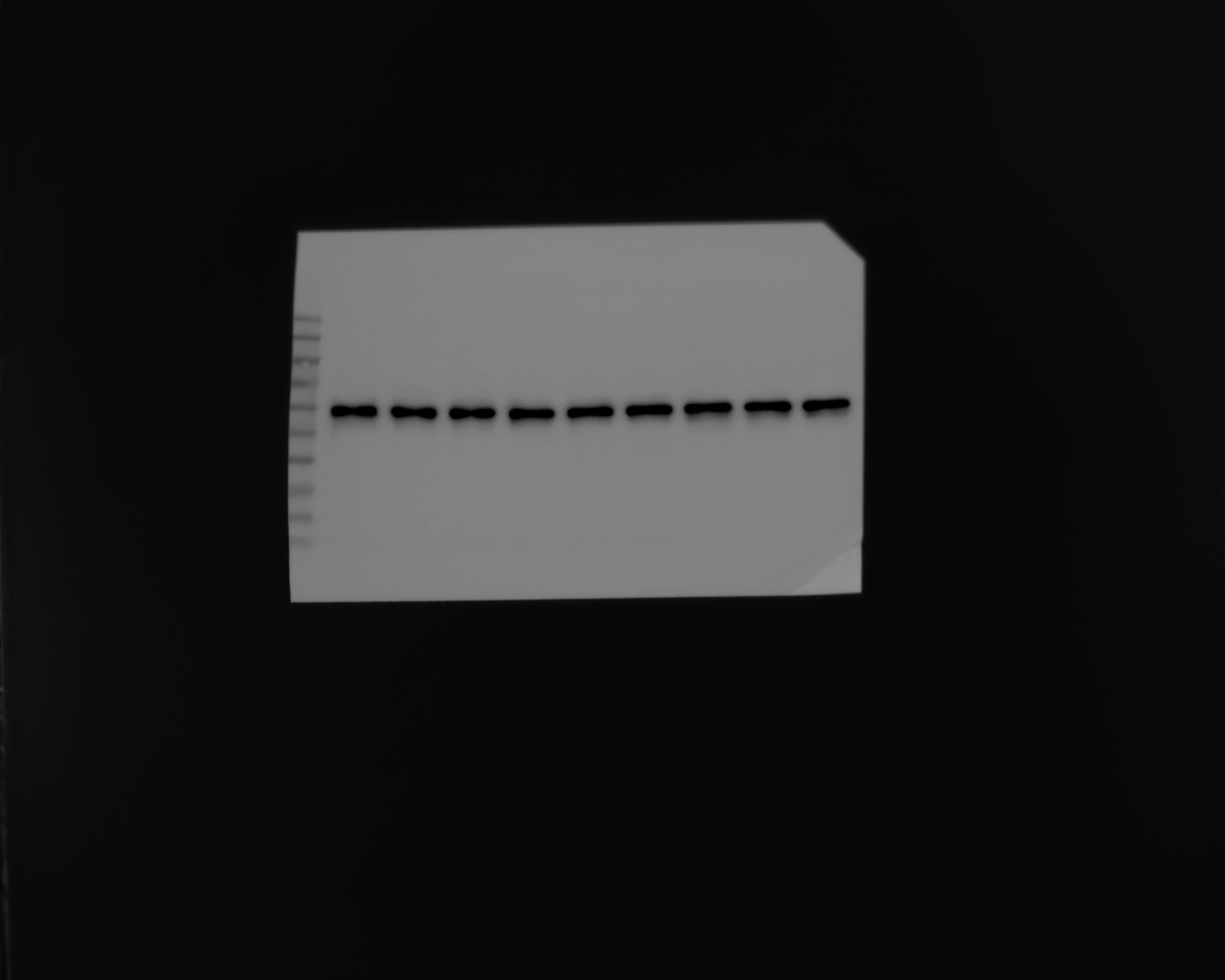

Supplement: Supplementary file 6 [file Image6.jpeg]

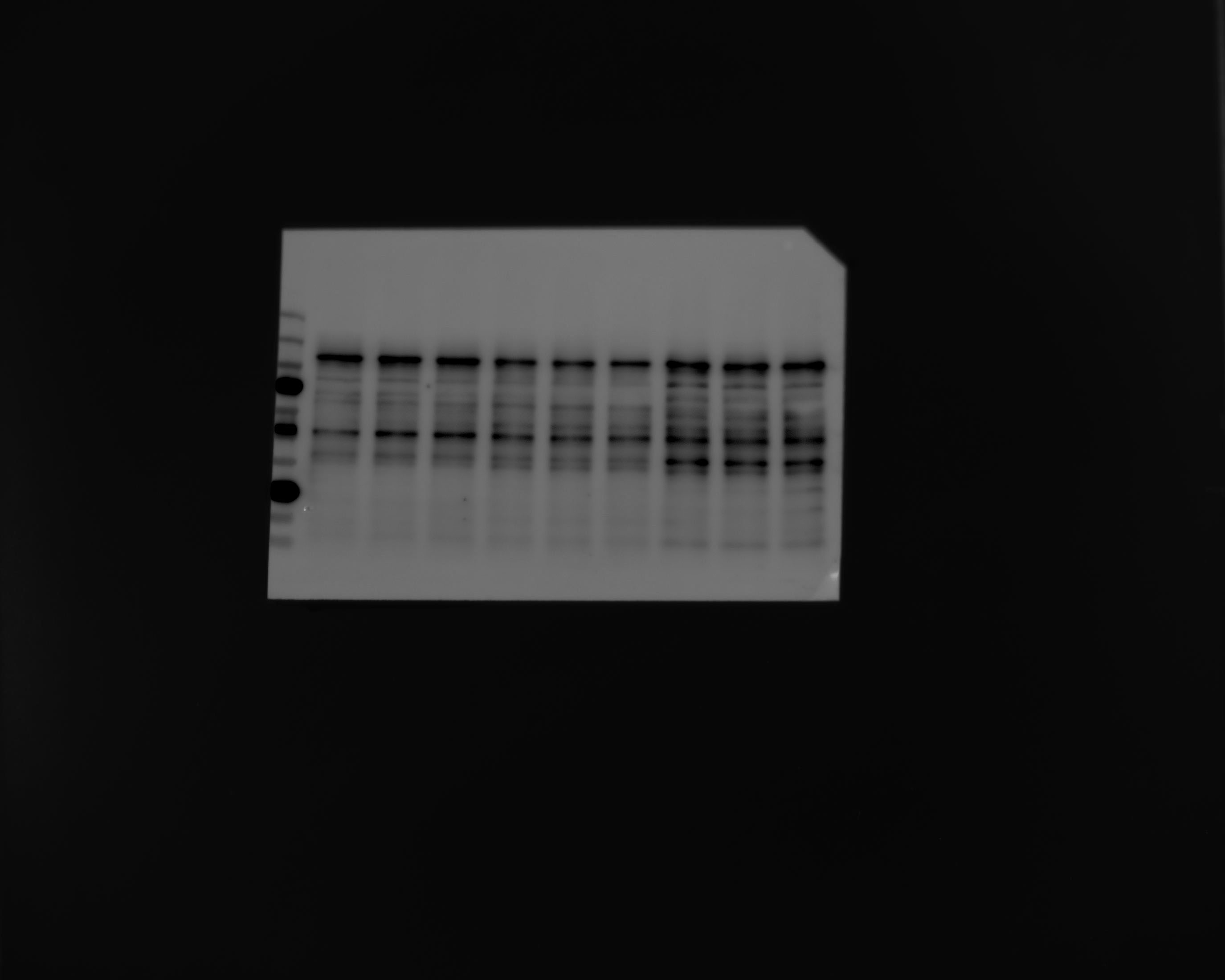

Supplement: Supplementary file 7 [file Image7.jpeg]

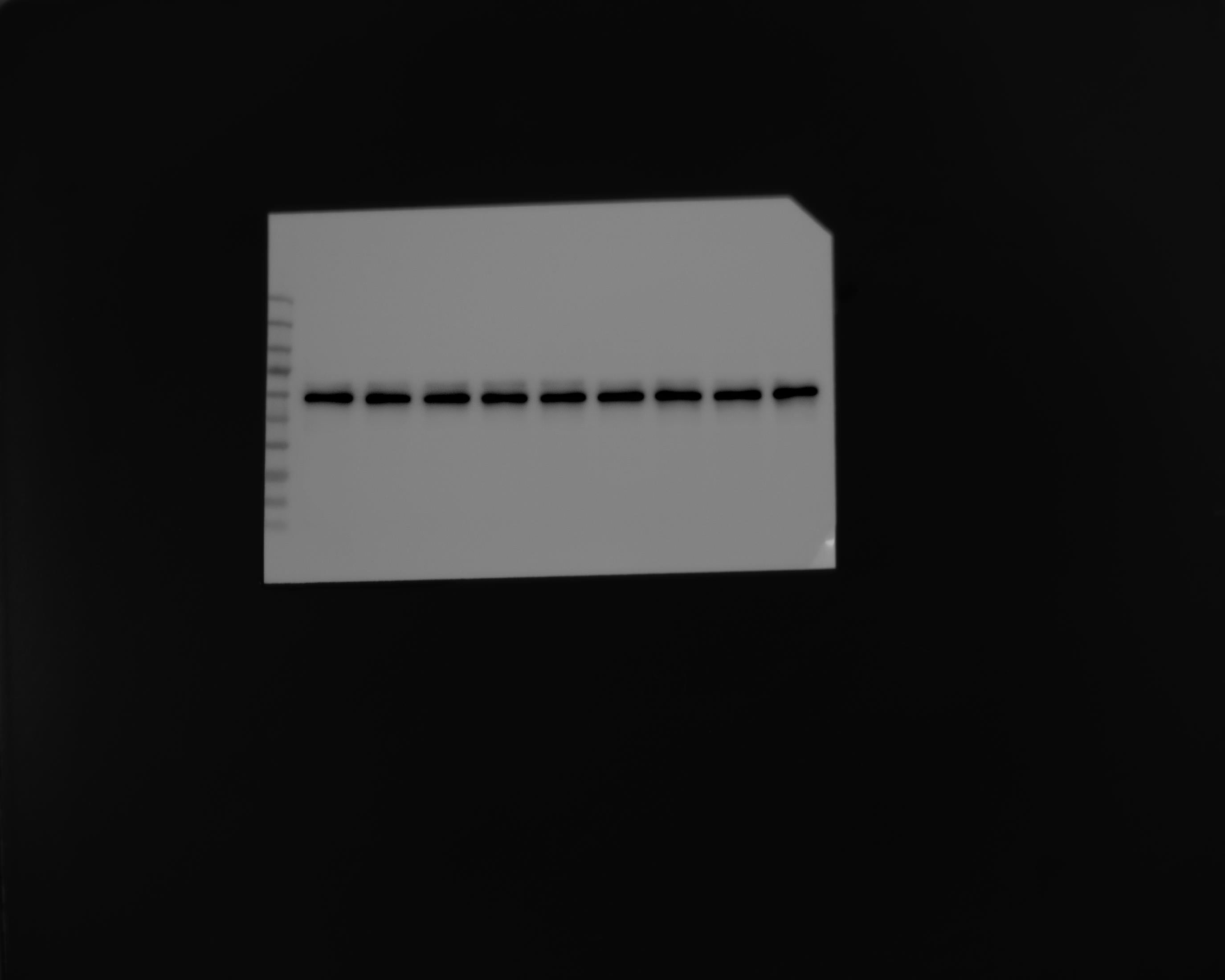

Supplement: Supplementary file 8 [file Image8.jpeg]

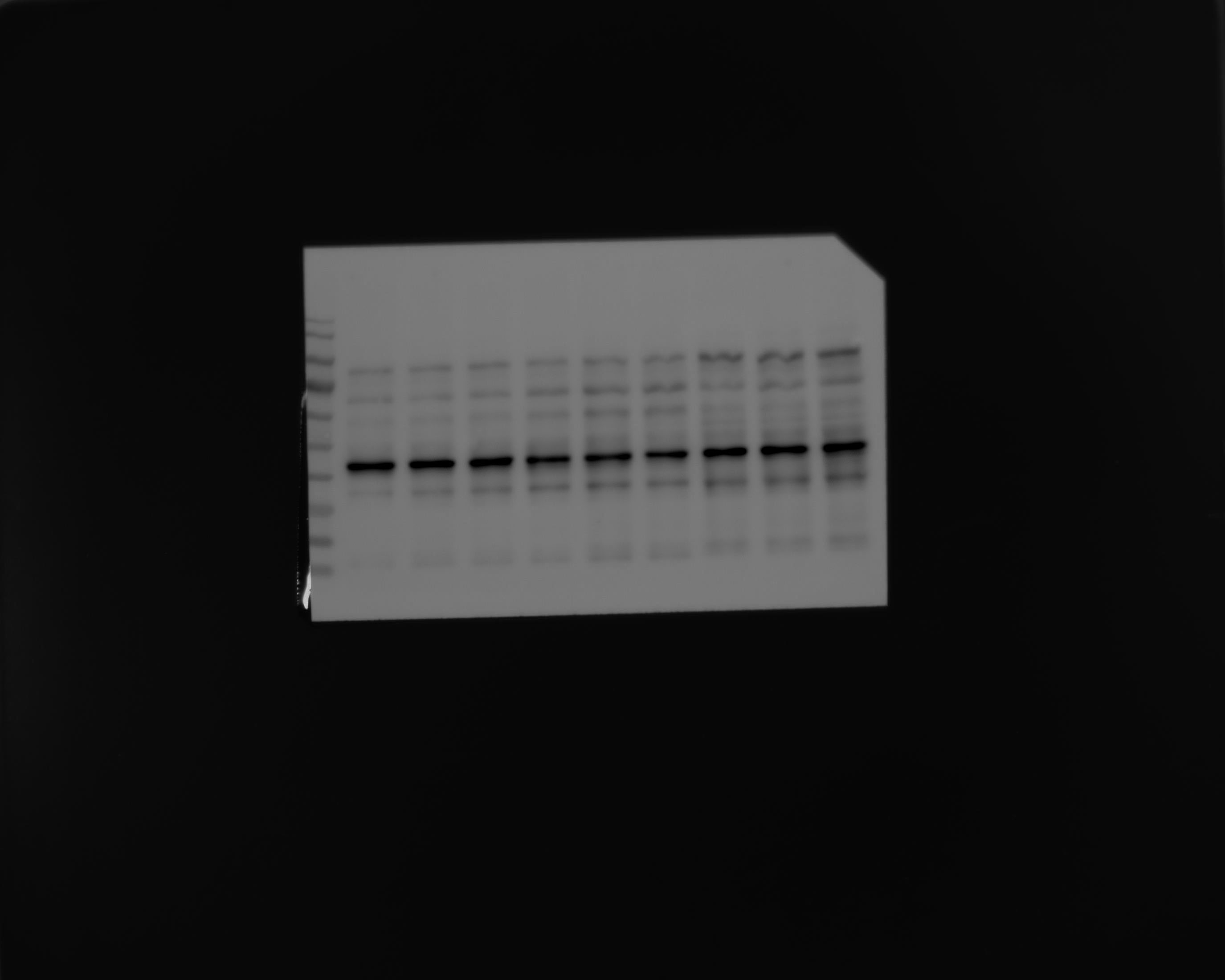

Supplement: Supplementary file 9 [file Image9.jpeg]

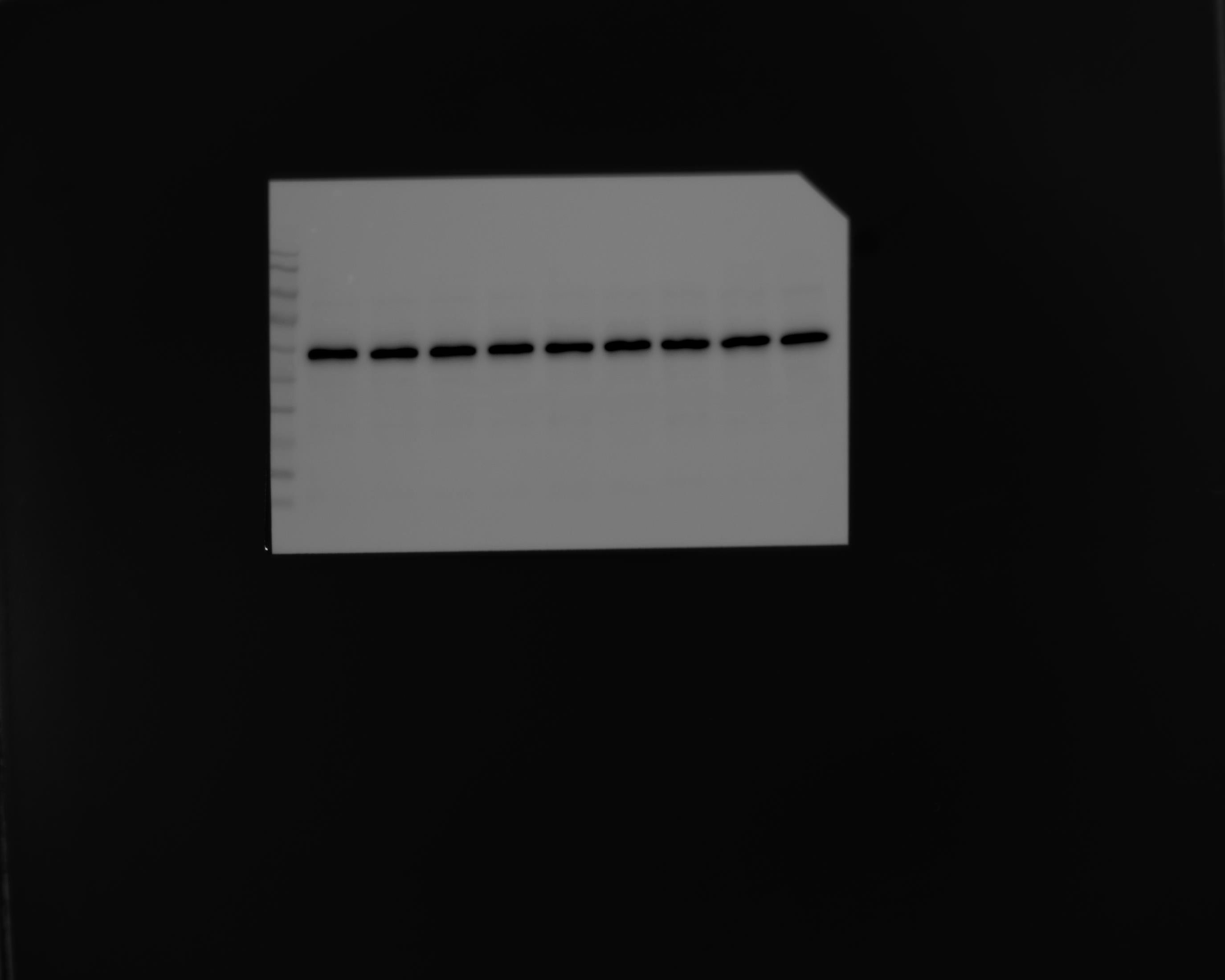

Supplement: Supplementary file 10 [file Image10.jpeg]

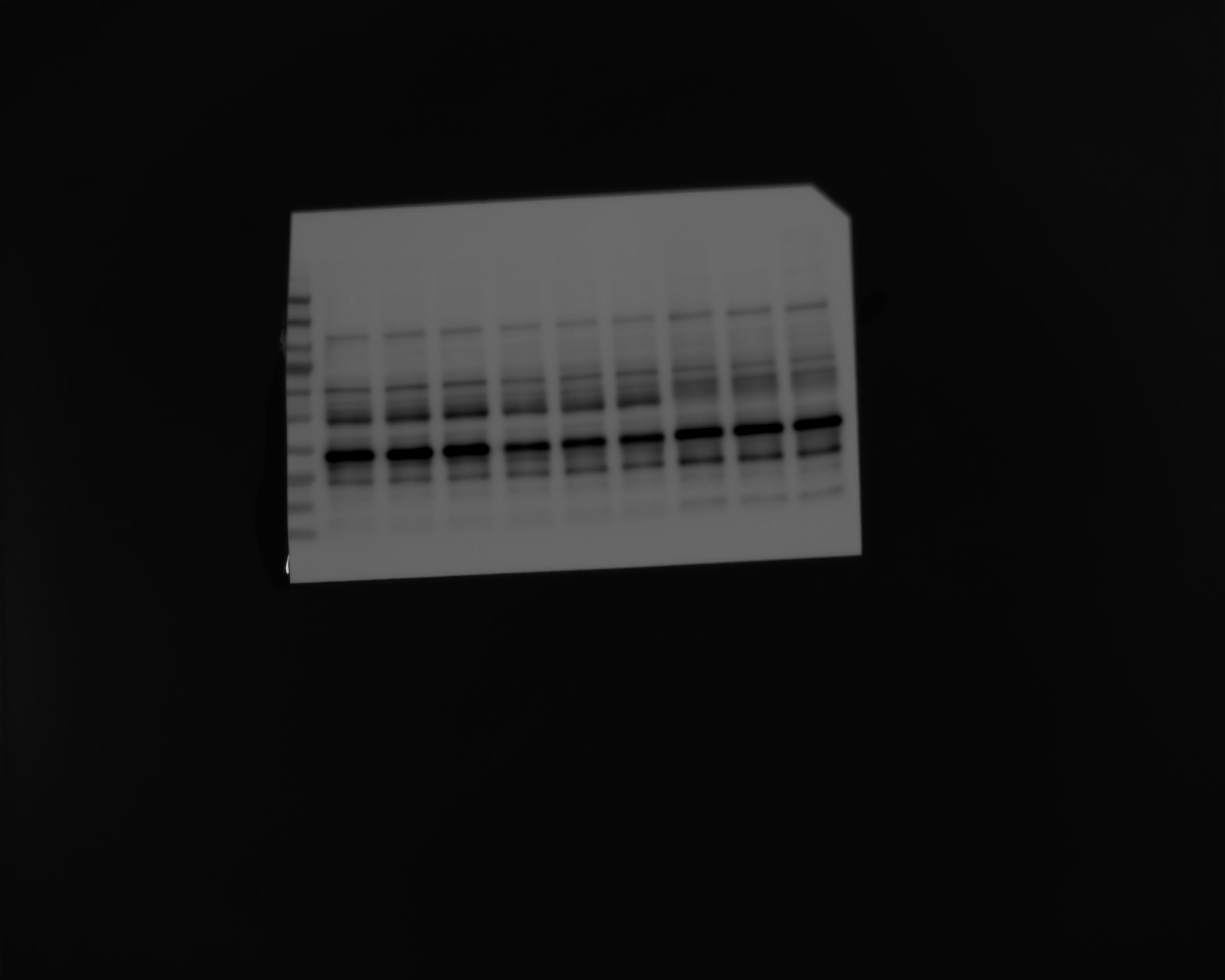

Supplement: Supplementary file 11 [file Image11.jpeg]

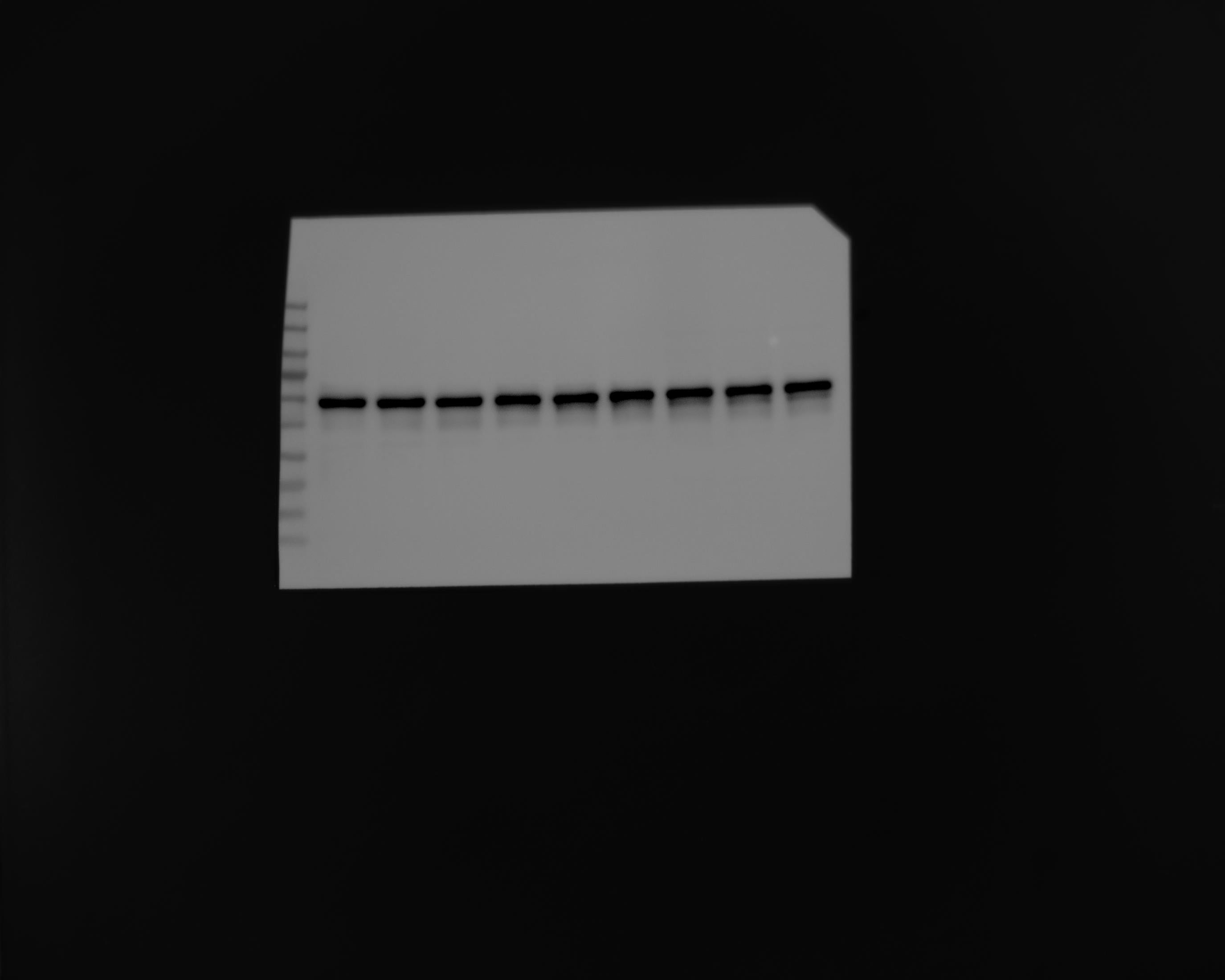

Supplement: Supplementary file 12 [file Image12.jpeg]
