# Supplementary material for: Transcriptomic elucidation of Dahuang-Huanglian in promoting white adipose browning in high-fat diet-induced obese rats
Source: Front Endocrinol (Lausanne). 2025 Oct 8;16:1652703. doi: 10.3389/fendo.2025.1652703 (PMC12540174; doi:10.3389/fendo.2025.1652703)
Supplement: Supplementary file 14 [file Table2.docx]

Supplementary Material

# Supplementary Figures and Tables

## Supplementary Tables

**DHHL improves body weight and blood glucose levels in obese rats**

As presented in Supplementary Table S2, after 12 weeks of DHHL treatment, both body weight and FBG were significantly elevated in the Model group compared to the Control group (*P* < 0.05). In contrast, DHHL administration notably reduced body weight and FBG levels in obese rats relative to the Model group (*P* < 0.05).

**Table S2 12 weeks body weight and FBG**

| Group | Drug dose (g/kg) | Body weight (g) | FBG (mmol/L) |
| --- | --- | --- | --- |
| Control | - | 406.76 ± 48.11 | 5.68 ± 0.72 |
| Model | - | 583.50 ± 59.52* | 7.34 ± 1.21* |
| Met | 0.18 | 511.49 ± 58.51^#^ | 5.64 ± 0.94^#^ |
| LDHHL | 0.9 | 560.51 ± 61.52 | 6.29 ± 1.10^#^ |
| MDHHL | 1.8 | 524.51 ± 57.50^#^ | 6.18 ± 1.04^#^ |
| HDHHL | 3.6 | 514.51 ± 53.49^#^ | 5.84 ± 0.86^#^ |

**P* < 0.05, vs. the Control group; ^#^*P*< 0.05, vs. the Model group

## Supplementary data

The data of transcriptome sequencing of white adipose tissue can be found at figshare.com (10.6084/m9.figshare.29162219).
